# Genome Wide Expression Profiling during Spinal Cord Regeneration Identifies Comprehensive Cellular Responses in Zebrafish

**DOI:** 10.1371/journal.pone.0084212

**Published:** 2014-01-20

**Authors:** Subhra Prakash Hui, Dhriti Sengupta, Serene Gek Ping Lee, Triparna Sen, Sudip Kundu, Sinnakaruppan Mathavan, Sukla Ghosh

**Affiliations:** 1 Department of Biophysics, Molecular Biology and Bioinformatics, University of Calcutta, Kolkata, India; 2 Genome Institute of Singapore, Singapore, Singapore; 3 Chittaranjan National Cancer Research Institute, Kolkata, India; Wayne State University School of Medicine, United States of America

## Abstract

**Background:**

Among the vertebrates, teleost and urodele amphibians are capable of regenerating their central nervous system. We have used zebrafish as a model to study spinal cord injury and regeneration. Relatively little is known about the molecular mechanisms underlying spinal cord regeneration and information based on high density oligonucleotide microarray was not available. We have used a high density microarray to profile the temporal transcriptome dynamics during the entire phenomenon.

**Results:**

A total of 3842 genes expressed differentially with significant fold changes during spinal cord regeneration. Cluster analysis revealed event specific dynamic expression of genes related to inflammation, cell death, cell migration, cell proliferation, neurogenesis, neural patterning and axonal regrowth. Spatio-temporal analysis of *stat3* expression suggested its possible function in controlling inflammation and cell proliferation. Genes involved in neurogenesis and their dorso-ventral patterning (*sox2* and *dbx2*) are differentially expressed. Injury induced cell proliferation is controlled by many cell cycle regulators and some are commonly expressed in regenerating fin, heart and retina. Expression pattern of certain pathway genes are identified for the first time during regeneration of spinal cord. Several genes involved in PNS regeneration in mammals like *stat3*, *socs3*, *atf3*, *mmp9* and *sox11* are upregulated in zebrafish SCI thus creating PNS like environment after injury.

**Conclusion:**

Our study provides a comprehensive genetic blue print of diverse cellular response(s) during regeneration of zebrafish spinal cord. The data highlights the importance of different event specific gene expression that could be better understood and manipulated further to induce successful regeneration in mammals.

## Introduction

Among the vertebrate, urodele amphibians and teleost fish have ability to regenerate their spinal cord after injury. In mammals following spinal cord injury (SCI) there are overwhelming inflammatory responses which trigger several other secondary tissue damage, neuronal and glial loss, progressive cavitation and glial scarring. These processes lead to functional decline and paralysis. Zebrafish is a powerful vertebrate model organism to elucidate gene function during regeneration, since they have extraordinary ability to regenerate their fins [1], [2], heart muscle [3] and central nervous system (CNS) [4]–[9] after injury. Adult zebrafish, in contrast to mammals, can re-grow axons readily after SCI and re-establish appropriate connections to recover significant functions [4], [6], [10]. In order to understand the mechanisms of inducing CNS regeneration in mammals, studies involving regeneration competent model organism is a prerequisite.

Regeneration of spinal cord has been studied by various groups using expression analysis of candidate genes or by generating transgenic zebrafish [11], [12]. However, parallel analysis of gene expression during different phases of regenerative events in spinal cord using high-density dedicated zebrafish arrays has not been attempted. In Medaka, another teleost fish, a small scale cDNA microarray screen during fin regeneration was reported using 2,900 expressed sequence tags (ESTs), which shared no homology to known genes [13]. Attempts have been made to employ Affymetrix arrays containing 14,900 transcripts representing 10,000 genes to study regeneration of fin, heart and retina in zebrafish [5], [14], [15]. High density arrays have been used to profile the transcriptome dynamics during embryogenesis [16] but such high-density microarray for genome-wide gene expression analysis has not previously been attempted for studying regeneration in adult zebrafish. For the present study, we have used a custom designed high-density oligonucleotide microarray for zebrafish (Agilent platform; 44000 probes) and determined the expression dynamics during the process of spinal cord regeneration at five different time points. We have tried to focus on the analysis of gene expression during different cellular events spanning all stages of regeneration. Previously we have characterized cellular events that include infiltration of blood cells, inflammation, cell death, proliferative response, neurogenesis and axonal regrowth during spinal cord regeneration in zebrafish [6]. Our present analysis, for the first time, reports a comprehensive genome wide expression analysis of regenerating zebrafish spinal cord after giving crush injury and we focused on event specific expression analysis during regeneration. We report here the genes that are differentially expressed in 5 different time points of regeneration and their involvement in the cellular events that modulate the process.

## Results and Discussion

Previously we have studied time course analysis of spinal cord regeneration after giving crush injury [6] which revealed that immediately after injury there is infiltration of various blood cells until 5 day post injury (dpi). Among them macrophage played a pivotal role in devouring and clearing apoptotic cells as well as myelin debris. Apoptotic cell death has been recognized very early within 6 hrs of injury and peaked around 24 hrs; subsequently it declined. Infiltration and cell death is followed by ependymal sealing and other regenerative response like migration and accumulation of cells near the injury epicenter. Injury induced proliferation is a key event after SCI in zebrafish [6], [12]. Proliferative response after injury is quite robust and initiated at 3 dpi cord, peaked at 7 dpi cord and later decreases gradually at 10 and 15 dpi cord. Ependymal cells are highly proliferative in nature and contribute to successful neurogenesis, which may include radial glia. Axonal regrowth can be seen in 15 dpi cord and remyelination is aided by the presence of Schwann cells. Both neurogenesis and axonogenesis is leading to successful regeneration in this species. We have studied the basic cellular events to uncover the molecular basis of some of the events characterized during spinal cord regeneration.

### Analysis of differentially expressed genes during spinal cord regeneration in zebrafish

To identify the underlying molecular events following crush injury, we have focused on gene expression profile during different stages of zebrafish spinal cord regeneration. The whole data represents gene expression in uninjured spinal cord (control) and injured spinal cord at five different time points of regeneration (Day 1, Day 3, Day 7, Day 10 and Day 15).

**Figure 1:**
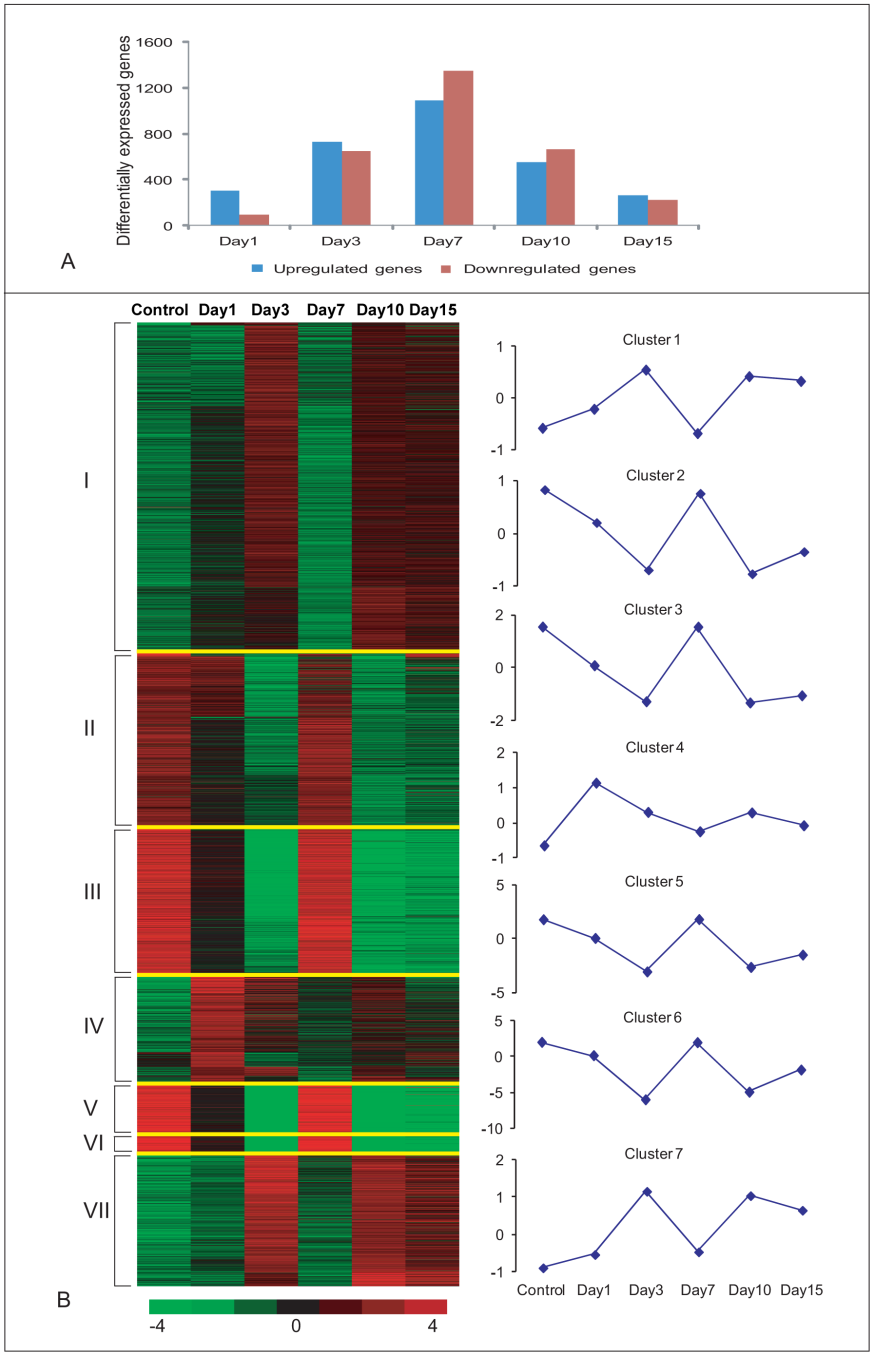
K-means Clustering Analysis of Differentially Expressed Genes before and after SCI in zebrafish. A) Distribution of differentially expressed genes both up-regulated and down-regulated after SCI at different time points. B) A set of 3,842 genes are identified as differentially expressed in at least one time point and are clustered into seven groups using the k-means algorithm. In the figure, horizontal lines indicate the expression pattern of each gene and the vertical columns indicate the time points after injury. The color chart indicates mean fold change of gene expression in each time points. Red and green colors represent increased and decreased expression respectively. Control = uninjured cord, Day 1 = 1 dpi cord, Day 3 = 3 dpi cord, Day 7 = 7 dpi cord, Day 10 = 10 dpi cord and Day 15 = 15 dpi cord. The clusters are separated by fine yellow lines. The average expression value of each cluster is plotted and shown in the line graph.

Following SAM and GeneSpring analysis of the microarray data, a total 3842 (fold change ≥2, FDR<0.05) differentially expressing unique genes were identified. From this unique set of annotated genes 304, 727, 1093, 551 and 263 genes are up-regulated at 1 dpi, 3 dpi, 7 dpi, 10 dpi and 15 dpi cords, respectively. On the other hand 93, 65, 1342, 666 and 226 genes are down-regulated at the above mentioned time points respectively (Fig. 1A, Table S1). Maximum numbers of genes are differentially expressed at 7 dpi cord (Fig. 1A). It is also evident that in early phases (Day 1 and Day 3) of regeneration, higher numbers of genes are up-regulated whereas in the later time point (Day 7 and Day 10) more genes are down-regulated. The differentially expressing genes at various points of regeneration are very dynamic in nature. Among the 3842 differentially expressed genes, as many as 2073 genes are up-regulated at certain stages of regeneration and down-regulated at other stages; whereas 864 and 905 genes are exclusively up-regulated and down-regulated, respectively.

The differentially expressed genes are clustered by using k-mean clustering algorithm (using the Cluster3.0). We have optimized ‘k’ = 7 (the selection of *k* was done using an R package for cluster validation named clValid) and number of iterations = 5000 for the genes expressed across the 5 time points of regeneration. Distinctly, 7 clusters are generated and are viewed using TreeView (Fig. 1B). The average value of each cluster is also displayed in the same figure. Cluster I and Cluster VII include genes that are maximally up-regulated in 3 dpi compared to other time points and consist of 1314 and 531 genes, respectively. The genes in cluster VII showed higher level of up or down regulation than Cluster I in all the time points. The genes in Cluster IV are unique (425 genes); these genes respond significantly immediately after injury (Day 1) and are subsequently down-regulated in all time points. The genes that are upregulated at 7 dpi are clustered in 4 groups viz. Cluster II, Cluster III, Cluster V and Cluster VI, containing 699, 586, 225 and 52 genes, respectively. Though these four clusters showed similar basic pattern of expression displaying maximum expression at 7 dpi compared to other stages of regeneration, the degree of average expression levels varied in these clusters (eg. Cluster VI from −5.9 to 2.0).

### Analysis of biological functional groups and enriched pathways

#### Cluster wise analysis of different functional groups

**Figure 2:**
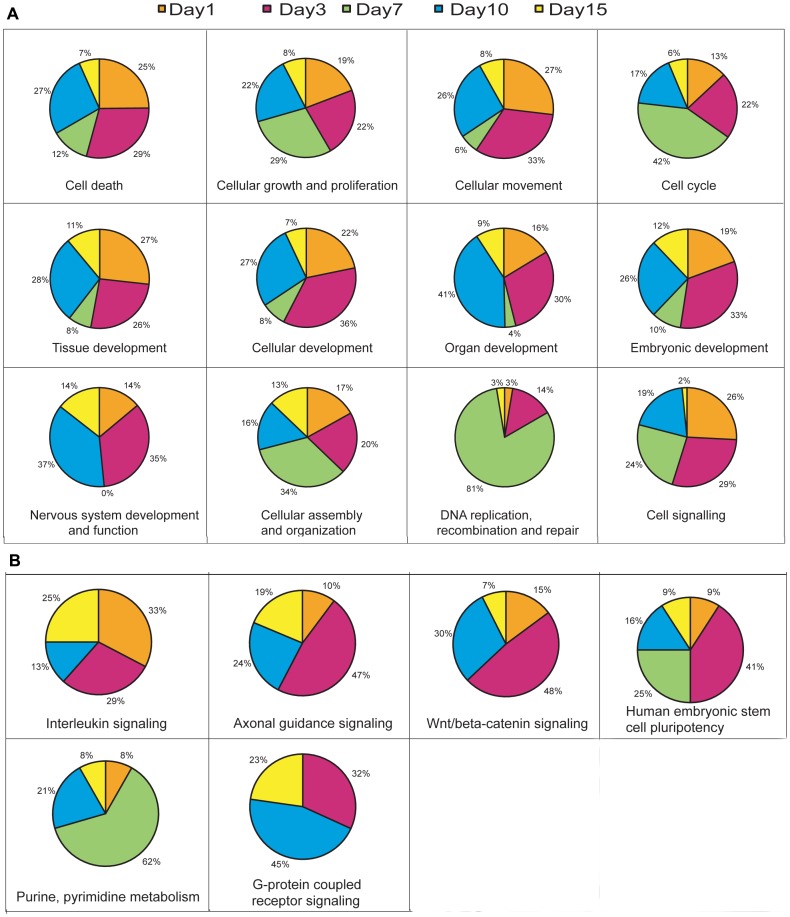
IPA analysis of biological functional groups and enriched pathways. A) Pie charts showing the percentage of genes that are differentially expressed (both up and down regulated) at different time points for the various enriched functional groups in IPA analysis. B) Pie charts showing the percentage of genes that are differentially expressed (both up and down regulated) for the various enriched signaling pathways in IPA analysis at different time points after SCI.

We intended to perform functional enrichment analysis on the clusters to study the functional groups that are largely activated during the process of regeneration. We have used Ingenuity Pathway Analysis (IPA) to identify functional groups. We provided both up and down-regulated genes of each cluster as input and selected 12 significantly enriched groups that are related to regeneration. All these groups are highly enriched in the clusters. Some of the enriched functions that we identified are ‘cell death’, ‘cellular growth and proliferation’, ‘cellular development’, ‘cell cycle’, ‘DNA replication and repair’, ‘nervous system development’ etc (Fig. 2A; Figure S1 and Table S2).

#### Functional group analysis in five different time points after injury

The set of differentially expressed genes, both up and down-regulated, are also grouped on the basis of their highest expression at a particular time point during regeneration. For instance, when a differentially expressing gene shows highest expression at 3 dpi, it is assigned to the 3 dpi group. Similarly, the whole data is divided into 5 groups (1, 3, 7, 10 and 15 dpi groups) and each group is subjected to IPA analysis. We observed that same set of functional groups are significantly enriched in these day-wise groups (Fig. 2A; Figure S2 and Table S2). To analyze the data in more detail and relate information to the different phases of regeneration, we have analyzed the functional enrichments during the stages of regeneration (Fig. 2A). It is evident that different functional groups are enriched in different time points suggesting their involvement with particular event(s) in regeneration. Early time points like 1 and 3 dpi showed enrichment of similar functional groups like ‘cell death’, ‘cell growth and proliferation’ and ‘cellular development’. Whereas in 7 dpi, which is the proliferative phase of regeneration, other functional groups like ‘cell cycle’, ‘cell growth and proliferation’, ‘DNA replication and repair’ are significantly enriched. Similarly, in late regenerative phase that is related to tissue repatterning, enriched functional groups are ‘nervous system development and function’, ‘tissue development’ and ‘organ development’.

IPA analysis also allowed us to identify enrichment of genes in canonical pathways during the process of regeneration. Some of the important canonical pathways that are enriched at different time points of regeneration are Wnt, axonal guidance, interleukin, purine-pyrimidine biosynthesis, G-protein coupled receptor signaling, human embryonic stem cell pluripotency signaling and several other signaling pathways (Fig. 2B; Figure S3 and Table S3). Interleukin (IL) signaling molecules are highly enriched in very early and late stages of regeneration and involved all different signaling pathways like Il-8, Il-6, Il-22, Il-10 and Il-k. Molecules related to axonal guidance signaling are highly enriched in 3 dpi, 10 dpi and 15 dpi cord, where highest enrichment is observed in 3 dpi cord. Embryonic stem cell pluripotency is enriched at several time points 3 dpi, 7 dpi and 10 dpi cord. We find a high predominance of molecules related to purine and pyrimidine metabolism in 7 dpi, when cell proliferation is at its highest peak. Similarly, cAMP mediated signaling is enriched in late time points, in 10 dpi and 15 dpi cord where axonal regeneration is initiated and maintained. Interestingly we observed that many signaling pathways are enriched in the initial time points (Day 1 and Day 3) and also in late time points (Day 10 and Day 15). Another salient observation is enrichment of few but very interesting pathways like ‘One Carbon Pool by Folate’ and ‘N-Glycan Biosynthesis’ at 7 dpi cord (Figure S3).

### Events involved in different phases of regeneration: Analysis of event specific genes

Regeneration of spinal cord involves highly coordinated cellular and molecular events. There are different phases like early regeneration phase, proliferative and growth phase and late regeneration phase. We have tried to identify genes involved in these different phases. The fold change and q-value for genes are provided in supporting information files.

Early regeneration phase involves events like inflammation, macrophage infiltration, cell death, cell migration and dedifferentiation. These events are initiated immediately after injury (0 hr-1day) and continue at least until Day 3. Immediately after injury there is infiltration of blood cells (predominantly macrophages) and a brief inflammatory response and cell death. The expression of different genes would reflect these specific events after injury.

#### Control of inflammatory response

**Figure 3:**
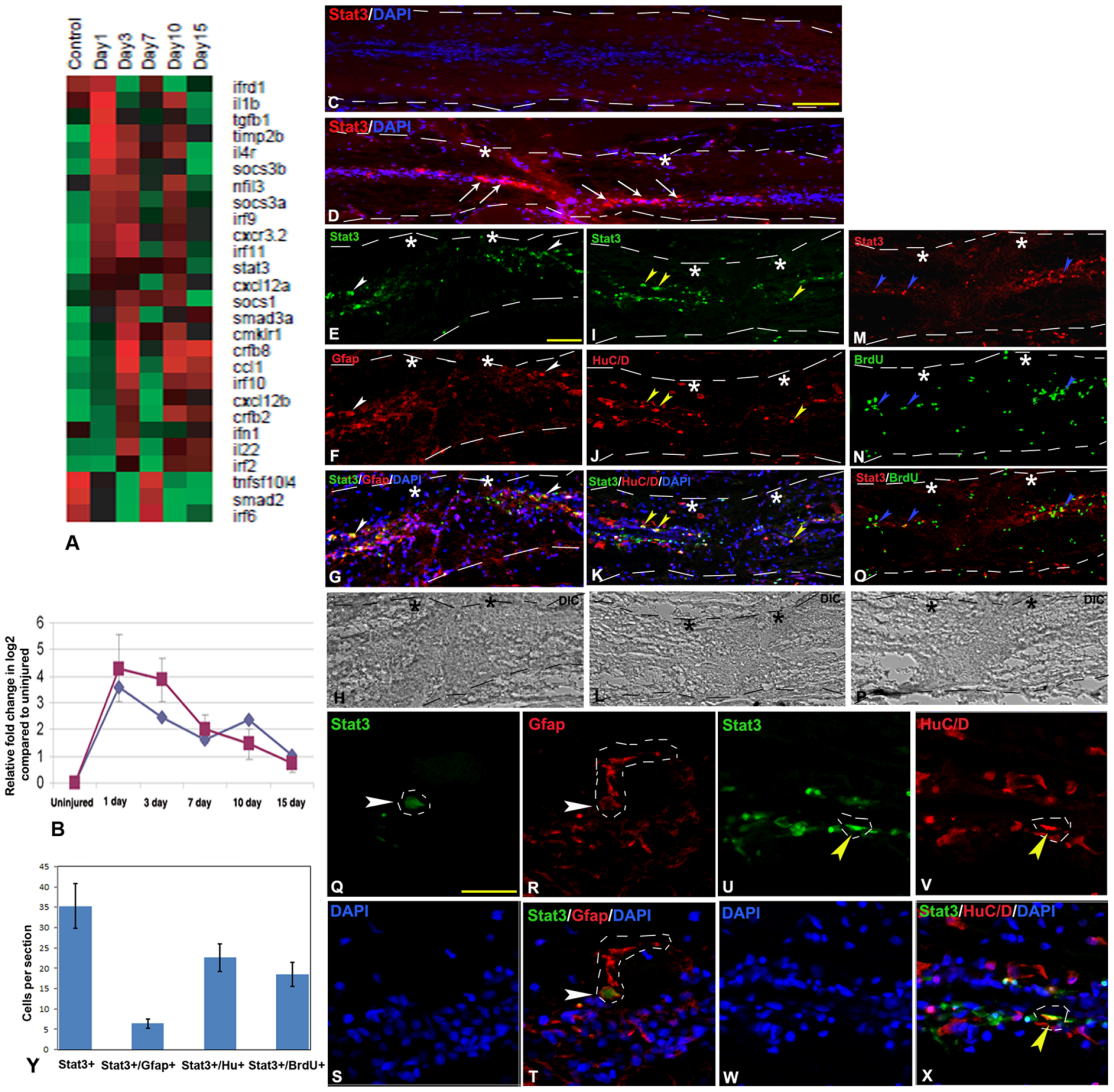
Differentially expressed genes related to inflammatory response and validation of *stat3* expression in uninjured and injured zebrafish spinal cord. A) Dendrogram representing genes related to regulation of inflammation. Each horizontal line indicates the expression pattern of each gene and the vertical columns indicate the uninjured control and time points after SCI. The color chart indicates mean fold change of gene expression in each time points. Red and green colors represent increased and decreased expression respectively. B) Quantitative RT-PCR of *stat3* expression showing fold change (red graph) and pattern of expression at different time points after injury. The temporal expression pattern of qRT-PCR (red graph) was compared with results of microarray analysis (blue graph). Error bars represent SEM, n = 3, p<0.01. C) A longitudinal section of uninjured cord stained with STAT3 and DAPI. D) A longitudinal section of 1 dpi cord showing many STAT3 positive cells (white arrows) close to the injury epicenter (double white stars) counter stained with DAPI. E–H) A longitudinal section of 3 dpi cord double stained with STAT3 and radial glial marker- GFAP, showing individual panels of STAT3 (E), GFAP (F), merge with DAPI (G) and DIC (Differential interference contrast) (H). A few STAT3^+^ cells are colocalized with GFAP^+^ cells (white arrowheads) close to injury epicenter. I–L) A longitudinal section of 3 dpi cord showing colocalization of STAT3 and newly formed neuronal marker, HuC/D with individual panels of STAT3 (I), HuC/D (J), merge with DAPI (K) and DIC (L). Some of the STAT3^+^ cells are colocalized with HuC/D^+^ cells (yellow arrowheads) close to the injury epicenter. M–P) A longitudinal section of 3 dpi cord colocalized with STAT3 and proliferating cell marker, BrdU shows individual panels of STAT3 (M), BrdU (N), merge (O) and DIC (P). Proliferating BrdU^+^ cells are also colocalized with STAT3^+^ cells (blue arrowheads) close to the injury epicenter. White dashed line in panel C to P mark the boundary of spinal cord tissue. Q–T) A representative higher magnification of 3 dpi cord section shows individual panels like STAT3 (Q), GFAP (R), DAPI (S) and merge (T). A STAT3^+^ cell (white arrowhead; the boundary of the cell nucleus is markedby white dashed line) is colocalized with GFAP^+^ radial glia (white arrowhead; the boundary of the cytoplasm of radial glia is marked by white dashed line). U–X) A representative higher magnification of 3 dpi cord section shows individual panels like STAT3 (U), HuC/D (V), DAPI (W) and merge (X). A STAT3^+^ cell (yellow arrowhead; the boundary of the cell nucleus is marked by white dashed line) is colocalized with HuC/D^+^ newly formed neuron close to ependyma (yellow arrowhead; the boundary of the cell nucleus is marked by white dashed line). Y) Quantitative analysis of STAT3^+^, STAT3^+^/GFAP^+^, STAT3^+^/HuC/D^+^ and STAT3^+^/BrdU^+^ cells in 3 dpi cord in longitudinal sections. The value represented as Mean±SEM of individual longitudinal section, n = 5 cord, p<0.01.Scale bar = 200 µm (C–D); 50 µm (E–P), 20 µm (Q–X).

**Figure 4:**
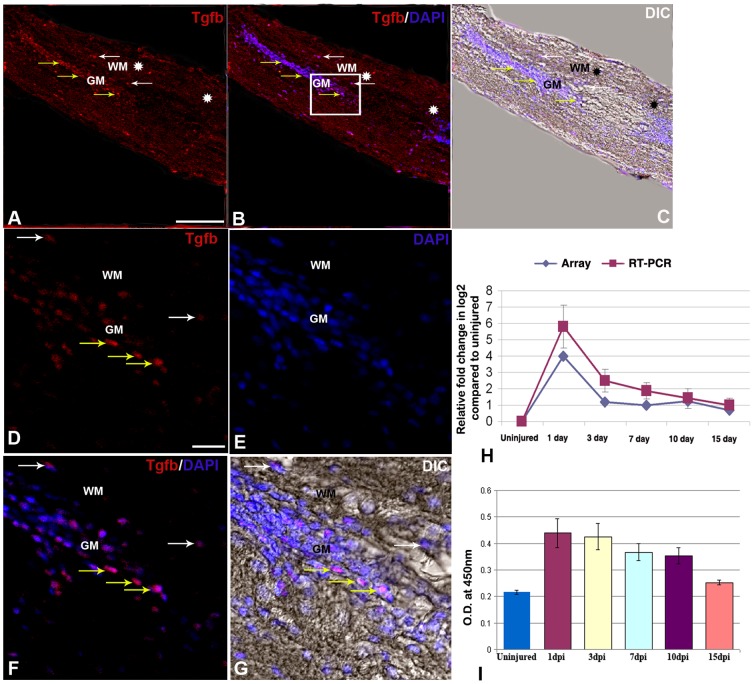
TGFβ expression in uninjured and injured zebrafish spinal cord. A) A longitudinal section of 1 dpi cord showing TGFβ positive cells in both grey matter (GM, yellow arrows) and white matter (WM, white arrows) of the cord at injury epicenter (double white star) and adjacent part. B) Same section merged with DAPI. C) Same section merged with DIC. D–G) Higher magnification of the boxed area of the section in B showing many TGFβ positive cells in both grey matter (GM, yellow arrows) and white matter (WM, white arrows) regions of the cord represented in individual panels of TGFβ (D), DAPI (E), merge of both (F) and merge with DIC (G). H) Quantitative RT-PCR of *tgbβ1* expression showing fold change (red graph) and pattern of expression at different time points after injury. The temporal expression pattern of qRT-PCR (red graph) was compared with results of microarray analysis (blue graph). Error bars represent SEM, n = 3, p<0.05. I) Quantification of TGFβ expression in uninjured and injured cord at different time points by ELISA. Error bars represent SEM, n = 5, p<0.01.Scale bar = 50 µm (A–C); 10 µm (D–G).

Inflammatory response is known to be controlled by members of cytokine, chemokine signaling pathways in mammalian SCI and other diseased state [17]–[19]. Here we found 27 differentially expressed genes (Fig. 3A, Table S4), among them, 21 genes were expressed their maximum level at 1 dpi or 3 dpi cord. Some of the differentially expressed important cytokines and chemokines are *ccl1*, *ccrl1a*, *cmklr1*, *crfb2*, *crfb8*, *cxcl12b*, *cxcr3.2*, *socs3a*, *socs3b*, *il1b*, *il4r*, *il22*, *irf10*, *irf11*, *irf8* and *irf9*. Molecules like *il4r*, *ifnα*, *tgfβ* are upregulated both in mammalian CNS [20] and in zebrafish spinal cord after injury as studied by us, but their temporal pattern and level of expression varies. For example, *tgfβ1* in mammalian brain after hypoxic injury shows 0.54 fold changes in 8 hr compared to 4 fold changes in 1 dpi zebrafish cord. On the contrary, *il10*, which is a neuroprotective interleukin, is downregulated in mammalian SCI [21] but upregulated in 1 dpi zebrafish cord (*il10* data not shown due to low fold change 1.36). The *tgfβ1* and *il10* play critical role in suppressing immune response [22], [23]. We observed that *socs3*, *stat3* and *tgfβ1* are highly upregulated in 1 dpi zebrafish cord that is in early phase of regeneration (Fig. 3 and 4, Table S4).

The array data of *stat3* and *tgfβ1* molecules have been further validated by qRT-PCR, immunohistochemistry and ELISA analysis (Fig. 3 and 4). Both *stat3* and *tgfβ1* show highest upregulation in 1 dpi cord and array hybridization data is confirmed by qRT-PCR, immunohistochemistry and ELISA analysis. STAT3 expression can be localized in injured cord, predominantly in the grey matter both in ependymal cells and in neuronal cells. It is important to mention that expression was observed exclusively at the injury site of 1 dpi and 3 dpi cord (Fig. 3D, E, I, M) and absent in the normal part of same cord. Colocalization study of STAT3 with GFAP (Fig. 3E–H, Q–T) and HuC/D (Fig. 3I–L, U–X) in 3 dpi cord suggests expression both in radial glia and in neurons, although we found that a higher number of STAT3 positive cells are HuC/D positive newly formed neurons [6] (Fig. 3K, X, Y; Table S5). We have also analyzed STAT3 colocalization with BrdU in 3 dpi cord, which is the beginning of proliferation stage and found that many STAT3^+^ cells are BrdU^+^ proliferating cells (Fig. 3M–P). There is also a population of STAT3 positive cells which are not colocalized with HuC/D and GFAP and they are probably inflammatory cells present in the injury epicenter of both 1 and 3 dpi cord. Our expression analysis of *tgfβ1* expression showed highest level of expression in 1 dpi cord and it was present in both grey and white matter of adjacent part of the epicenter (Fig. 4A–G). TGFβ, a multi-potent cytokine, plays critical role in suppressing the immune response after CNS injury in mammals [23]–[26]. Furthermore, it also plays a role in epithelial-mesenchymal transition (EMT) after SCI, expressed in M2 type macrophages [27] and may induce initiation of cell proliferation during tail regeneration in *Xenopus* [28].

Other analysis in mammalian CNS injury refers to importance of complement factors and MHC class I and II molecules expression [20], [29]. We observed upregulation of two complement factors *c1qc* and *c1a13b* early after injury (Table S4). Between these two factors prominent role of *c1qc* in activation of phagocytosis of macrophage/microglia in mammalian CNS is well known [30] which may suggest a similar role in zebrafish SCI where we observe increased phagocytosis by macrophage in 3 dpi cord [6]. Unlike mammalian CNS injury, where both MHC class I and II molecules are upregulated, we only found upregulation of MHC class I molecules like *mhc1uba*, *mhc1uaa* and *mhc1ze* in early time points in zebrafish cord regeneration (Table S4).

#### Pro- and anti-inflammatory macrophages

Our previous study highlighted importance of macrophage during early phase of regeneration in zebrafish spinal cord. In mammalian SCI presence of both pro- and anti- inflammatory macrophages are related to neuroprotective and neurodegenerative response [27]. In this context, it is worth mentioning that M1 macrophages are pro-inflammatory as characterized by pro-inflammatory cytokine release [31] and M2 macrophages as characterized by presence of scavenger receptors, IL-1 receptor antagonist [32] resulting in decreased production of pro-inflammatory cytokines like *il-1β* and upregulation of anti-inflammatory cytokines like *il-4* and *il-13* [27], [33]. We observed presence of a higher number of M2 type macrophage related genes (anti-inflammatory) compared to M1 type (pro-inflammatory) (Table S6). M2 type macrophage related genes include *scarb1*, *scarb2*, *il4r*, *tgfβ1*, *vegfa*, *tgif1*, *arg2* and many of them are upregulated in 1 dpi and 3 dpi cord.. Only two of the genes related to M1 type macrophage (*caspa* and *nos2*) are expressed. Suppression of *nos2* limit self-damage of phagocytic macrophages [34]. Similarly, we also found *nos2* expression as a consequence of inflammatory response.

Activated macrophage are known to play a role in wound healing, allergic reaction and proliferation and arginase activity controls the role of activated macrophages after injury [35]–[37]. Inflammatory response is complex as we observe both pro- and anti-inflammatory types of molecules, although a bias towards anti-inflammatory response was observed as we see strong upregulation of many M2 type macrophage related molecules. Macrophage may also enhance axonal growth by secreting several molecules like cytokines and neurotrophic factors and allowing Schwann cell migration as it happens in peripheral nervous system (PNS) [38]. Macrophage along with Schwann cell helps in clearing myelin and axonal debris as reported by others and us [6], [39]–[41]. Macrophage may also induce MMP-9, which is playing important role in debris clearance and preventing collagen scar formation [42], [43]. Our present data highlight the importance of macrophage as a key player in controlling inflammation, debris clearance and creation of permissive environment for axonal regrowth during regeneration.

#### Cell death

In mammalian SCI cell death contributes to huge neuronal and glial loss and involves distinct necrotic and apoptotic pathways. Apoptotic cell death is common to both mammalian and zebrafish SCI but in a different spatio-temporal pattern [6], [44]. We have identified many cell death related genes, their regulators and anti-apoptotic genes with differential expression during regeneration (Table S7). Two members, *tnfα* induced protein and *fasl*, which are upregulated in different time points and may have dual role in controlling apoptotic and necrotic pathway. Apoptotic cell death in mammalian CNS injury is regulated by Caspase and Calpain family of genes [45], [46] and we observed upregulation of *caspa*, *caspb*, *casp7*, *capn9* in 1 dpi and 3 dpi zebrafish cord. Upregulation of 12 apoptotic genes in early time points like 1 dpi and 3 dpi are probably induced as a consequence of injury, as these genes are absent in control, and are significantly downregulated in 7 dpi cord. Among these 12 genes, *capn9* and *traf4a* are upregulated in 10 dpi and 15 dpi cord. Significance of expression of these molecules in late time points during regeneration is not well understood although these are implicated in zebrafish CNS development [47]. Mammalian anti-apoptotic protein Bcl-X_L_ is known to control cell death and cell survival [48]. We also observed that many anti-apoptotic genes like *bcl2113*, *mcm5*, *bag3*, *tnfaip6* and *igf1ra* [49]–[52] are upregulated in different time points after injury.

#### Cell migration and epithelial-mesenchymal transition

Cell migration is one of the key phenomena in early regeneration time points as we showed that accumulation of cells in the injury epicenter occur as early as Day 3 and continues at least until Day 7 post injury [6]. Molecules like fibronectin, integrin, laminin, cadherin, matrix metalloproteinase(s) (MMPs) and collagen are known to be involved in cell adhesion, cell migration in developing and regenerating organs [5], [14], [15], [53]–[57]. Our data showed that most of the genes related to cell migration are up-regulated either in 1 dpi (*fn1b*, *mmp13*, *mmp9*) or in 3 dpi (*itgav*, *vim*, *itgb4*, *itgb3a*, *cdh2*, *cdh23*, *lamb4*, *lamb1*, *cxcl12b*) cord (Table S8). Many genes are also upregulated in different regenerating organs probably indicate their common role in tissue remodeling [15], [54]–[56]. Epithelial-mesenchymal transition (EMT) or interaction during regeneration is well known [54], [58], [59]. A similar EMT is probably operating during early phases of regenerating zebrafish spinal cord although it has not been characterized yet. We see expression of 8 EMT related genes which are upregulated in zebrafish cord after SCI (Table S8). A separate group of six genes represents Adam and Integrins of which *adam8a* and *adam9* show their upregulation in early time point (Table S8). Genes like *cdh2*, *cdh23*, *lamb4*, *lamb1*, *cxcl12b* are highly upregulated in 3 dpi cord. Further upregulation of these genes at 10 dpi or at 15 dpi cord, may suggest their role in different types of cell migration. In this context, it is important to refer that both neuronal migration and Schwann cell migration [60], [61] is known to occur at later time points after injury.

Epithelial-mesnchymal (EM) interaction is a phenomenon during dedifferentiation phase. We see expression of some of the genes related to dedifferentiation process (like *msx-c*, *msx-e* and *vim*) in 3 dpi zebrafish cord (Table S9).

#### Proliferative response after injury in spinal cord

**Figure 5:**
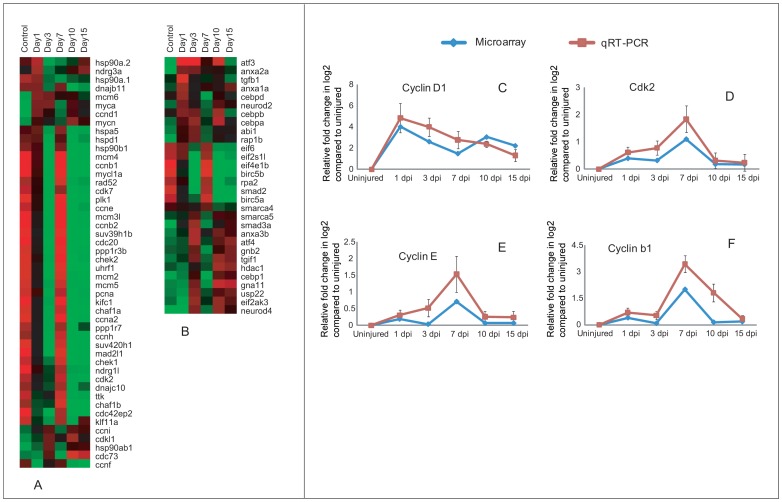
Differentially expressed genes related to cell proliferation in zebrafish spinal cord after injury and validation of cell cycle regulators. A) Dendrogram representing a group of cell cycle regulators directly involved with different cell cycle phases. B) Same representing group of genes indirectly control cell proliferation which include both negative and positive regulators. Each horizontal line indicates the expression pattern of each gene and the vertical columns indicate the uninjured control and time points after SCI. The color chart indicates mean fold change of gene expression in each time points. Red and green colors represent increased and decreased expression respectively. C–F) Quantitative RT-PCR analysis of *ccnd1* (C), *cdk2* (D), *ccne* (E) and *ccnb1* (F) showing fold change and pattern of expression at different time points after injury. The temporal expression pattern of qRT-PCR (red graph) was compared with results of microarray analysis (blue graph). Error bars represent SEM, n = 3, p<0.05.

Injury induced proliferation has been documented previously and proliferation is necessary to replace the lost neural cells. The proliferative response is controlled by many genes, which have been categorized as genes related to cell cycle and other regulators, involved in proliferation (Fig. 5; Table S10). As many as 48 genes associated with cell cycle are differentially regulated. Interestingly almost all genes expressed in uninjured control are also represented in 7 dpi cord, but are not expressed in other injury time points. Exception among these patterns is *hsp90a.1* and *hsp90a.2*, which are upregulated in uninjured cord and 1 dpi but down regulated in other injury time points (Fig. 5A). A high number of genes are Cyclins and Cdc/Cdks (Fig. 5A; Table S10). Based on cell counts of colocalized BrdU/H3P along with DAPI indicate that in uninjured cord there are a low number of cells in S-phase (2%) and M-phase (1%) compared to Go-G1 phase (97%). Number of cells in both S-phase and M-phase, increases after injury, although percentage of cells entering into S-phase are higher in both 3 dpi and 7 dpi cord (8% and 12% respectively) when compared to percentage of cells in M-phase (2% and 5% respectively) (Figure S4). Our present data on array analysis corroborated with the cell count data and showed that genes involved in G1-S phase transition are selectively upregulated either in 3 dpi (*ccnd1*, *ccni* and *myca*) and 7 dpi (*cdk2*, *cdk7*, *ccne* and *ccnh*) cord and all 3 genes associated with S-phase (*ccna2*, *pcna*, *uhrf1*) are upregulated only in 7 dpi cord when highest number of proliferating cells are in S-phase. Both the uninjured and injured cord showed expression of *cdk2*, *ccnh*, *ccne* and *ccna2*, but fold change varies, expression level is higher in injured than uninjured cord. Among 12 genes involved in G2-M transition, 10 genes (*ccnb1*, *ccnb2*, *cdc20*, *klf11a*, *mcm6*, *mcm2*, *mad2l1*, *ttk*, *plk1*, *kifc1*) which are M-phase related are expressed in both uninjured control and higher in 7 dpi cord. Expression of four genes have been further validated by qRT-PCR which confirms upregulation of *ccnd1* within 1 dpi to 3 dpi cord and *ccne*, *cdk2* and *ccnb1* in 7 dpi cord (Fig. 5C–F). Cell cycle regulators like *myca* and *ccnd1* both are down regulated in uninjured cord but upregulated early after injury and *myca* is known to upregulate its downstream molecules *ccnd1* and *cdk2* [62]. Cell cycle regulators like *cdc20* have a role in degradation of *ccnb1* during mitosis [63]; both *cdc20* and *ccnb1* are expressed in uninjured cord and highly upregulated in 7 dpi cord and subsequent downregulation of *ccnb1* in 10–15 dpi cord probably refers to reduction of cell proliferation and down regulation of mitosis markers at late stage of regeneration [6]. Cell cycle regulators also include members of *Mcm* families, members of *Hsp* families, *plk1*, *ttk*, *klf11a*, *ppp1r3b* and *ppp1r7* (approximately 19 genes) are upregulated both in uninjured control and 7 dpi cord and many of them are M-phase regulators.

As mentioned earlier that proliferative response are being controlled by cell cycle regulators along with a second category of genes, which include cell proliferation related genes (Fig. 5B; Table S10). 30 genes are further separated into four different subgroups based on their temporal expression pattern and known function. There are 11 genes, which are known to be negative regulators of cell cycle progression, mostly upregulated in 1 dpi and 3 dpi cords. Among them *anxa1a*, *cebpd* and *cebpb* are all upregulated in 1 dpi cord and may play a role in arresting of G1-S transition [64], [65]. Another important functional group of genes involved in positive regulation of cell cycle progression includes 12 genes. *Atf-3* is one of the highly upregulated genes in 1 dpi and 3 dpi cord compared to uninjured cord. A similar elevated expression of *atf-3* was demonstrated after injury in optic nerve [66], in dorsal root ganglia (DRG) and in motor neurons [67].

#### Differentially expressed genes involved in neurogenesis and neuronal differentiation

**Figure 6:**
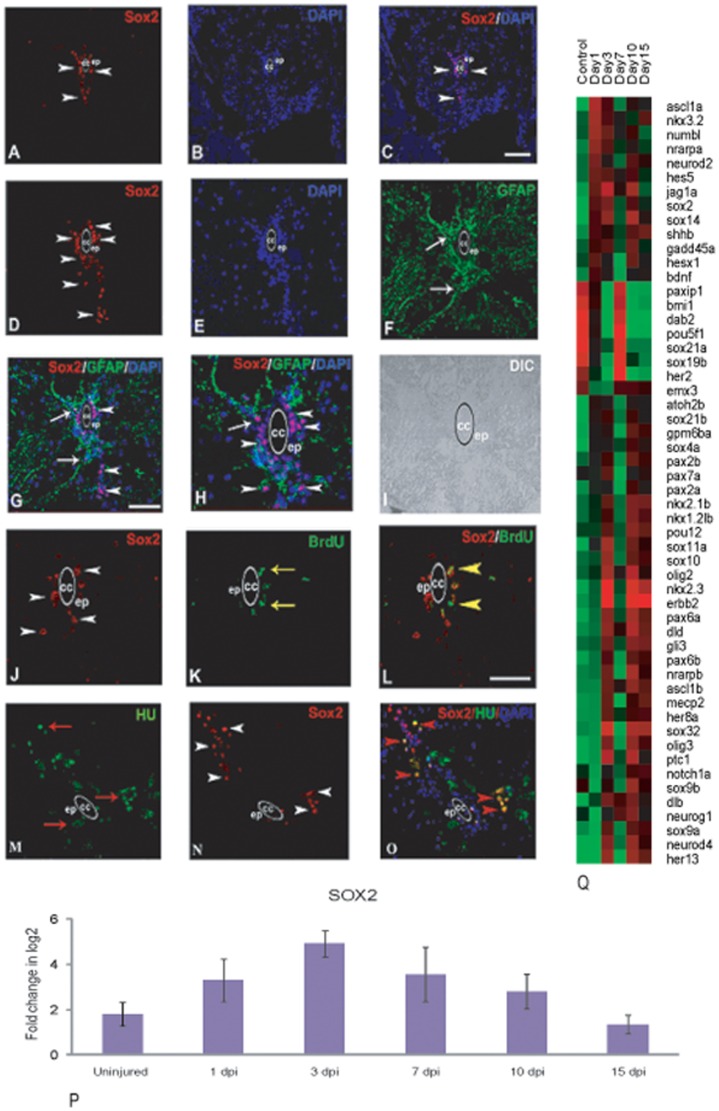
Differentially expressed genes involved in neurogenesis and neuronal differentiation after injury in zebrafish spinal cord and validation of Sox2 expression by immunohistochemistry and qRT-PCR analysis. A–C) A cross section of uninjured cord showing SOX2 positive cells (white arrowhead) in ependyma (ep) around the central canal (cc), counter stained with DAPI (B) and merge (C). D) A cross section of 3 dpi cord showing higher number of SOX2 positive cells compared to uninjured (A) in grey matter (white arrowhead) mostly in ependyma (ep) around the central canal (cc). E–F) Same 3 dpi cord section counter stained with DAPI (E) and stained with radial glial marker GFAP (F). G) Same 3 dpi cord section showing both SOX2 positive cells (arrowheads) and their colocalization with GFAP (arrow). H) Same section (G) in higher magnification showing colocalized SOX2 and GFAP positive cells (arrow) around the central canal (cc), I) DIC of same section in H. J–L) 3 dpi cord section showing SOX2 positive cells (J, arrowheads) and BrdU positive cells (K, yellow arrow) in the grey matter and many of them are SOX2^+^/BrdU^+^ (L, yellow arrowheads) in ependyma (ep) around the central canal (cc). M–O) A 3 dpi cord section showing many HuC/D positive neuronal cells (M, red arrow) and SOX2 positive cells (N, white arrowheads) in the grey matter. Same section showing colocalization of SOX2 and HuC/D (O, red arrowheads). P) qRT-PCR of *sox2* in uninjured cord and in injured cord at different time points. Error bars represent SEM, n = 3, p<0.05. Q) Dendrogram showing differentially expressed genes involved in neurogenesis and neuronal differentiation after injury. Each horizontal line indicates the expression pattern of each gene and the vertical columns indicate the uninjured control and time points after SCI. The color chart indicates mean fold change of gene expression in each time points. Red and green colors represent increased and decreased expression respectively. Scale bar = 50 µm (A–G and M–O), 20 µm (H–L).

Many genes related to neurogenesis are differentially regulated during the process of regeneration. Previously we showed that generation of new neurons is related to cell proliferation and Hu^+^/Brdu^+^ cells are continuously present in 3 dpi, 7 dpi and 10 dpi cord. Some of these cells become NeuroD positive and have undergone full neuronal differentiation [6]. Molecular analysis showed at least 54 genes are differentially expressed (Fig. 6Q; Table S11) and among them 41 genes are transcription factors (Figure S5). All the 54 genes are categorized in different groups based on their temporal pattern of expression. Group-I includes *her2*, *dab2*, *pou5f1*, *emx3*, *bmi*, *paxip*, *sox19b* and *sox21a* that are expressed in uninjured control and are upregulated in 7 dpi cord, when we also observe a very high level of proliferation, which contributes significantly to neurogenesis [6]. Among these genes, expressions of *her2* and *dab2* have been reported during retinal neurogenesis and in proliferating neuroepithelium of developing CNS [68], [69]. Group-II consists of large number of genes (36 genes) that are upregulated in 3 dpi, 10 dpi and 15 dpi cord and down regulated in uninjured control and 7 dpi cord. These are injury induced since these are not expressed in uninjured control. Genes like *notch1a*, *jagg1a*, *deltab* and *deltad* are all upregulated in 3 dpi and 10 dpi cord whereas proneural genes like *neurog1*, *neurod2*, *neurod4* and *olig2* are all upregulated in 10 dpi cord. Many of the genes in this group are members of Notch signaling pathway. Similar to developing CNS the above mentioned genes may promote differentiation of progenitors selectively to different neural fate and maintenance of reserve pools. Group-III consists of 10 genes *neurod2*, *nrarpa*, *hes5*, *nkx3.2*, *ascl1a*, *numb1*, *hesx*, *shhb*, *bdnf* and *gadd45a* all of these are highly upregulated in 1 dpi but downregulated in uninjured cord.

Many members of Sox gene family are differentially controlled during neurogenesis. There are two distinct pattern of expression within Sox family members. *Sox19b* and *sox21a* are upregulated both in uninjured cord and in 7 dpi cord. Others Sox family members (*sox2*, *sox4a*, *sox9a*, *sox9b*, *sox10*, *sox11a*, *sox14*, *sox21b* and *sox32*) are injury induced since they are upregulated in 3 dpi, 10 dpi and 15 dpi cord but down regulated in uninjured and 7 dpi cord. The highest expression values are different for different *sox* genes suggesting multiple functions during regeneration. Sox2, one of the key transcription factors is expressed in the neuroepithelium of the developing mammalian CNS and adult zebrafish brain. Its function in neural stem cell (NSC) maintenance, proliferation and specifying their identity [70]–[73] is well known. Our real-time PCR analysis showed that there is upregulation of *sox2* after injury at Day 3 compared to uninjured cord (Fig. 6P). The immunohistochemical analysis showed that in uninjured cord SOX2 positive cells are present both in ventricular region and in subependymal region of grey matter (Fig. 6A–C). A higher number of SOX2 positive cells have been found in the grey matter of 3 dpi cord (Fig. 6D–H) compared to uninjured cord. These cells are situated mostly in ependyma and few are in subependyma. A subpopulation of SOX2 positive cells are radial glia since they co-localize with GFAP with appropriate morphology (Fig. 6G–H; Table S12). When we co-localize SOX2 positive cells with a neuronal marker (HuC/D), we found that many newly formed HuC/D^+^ neuronal cells (as they are morphologically smaller than specified mature neuron) in the grey matter are SOX2 positive (Fig. 6M–O; Table S12). The colocalization of SOX2 with BrdU identified many cells present in both ventricular and subventricular region (Fig. 6J–L; Table S12) thus confirming presence of proliferating neural progenitors in regenerating cord.

#### Differentially expressed genes involved in repatterning

**Figure 7:**
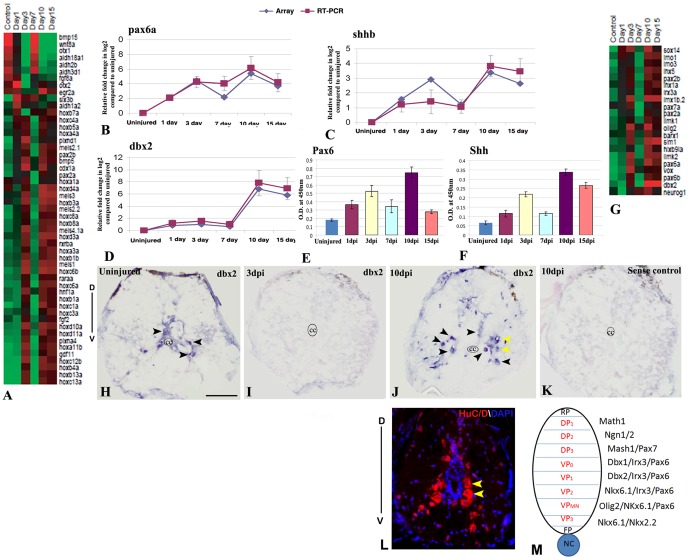
Differential expression of pattern forming genes during zebrafish spinal cord regeneration and validations of *pax6a*, *shhb* and *dbx2*. A) Represents known genes related to anterio-posterior patterning in CNS that are differentially expressed at different time points after SCI. The color chart indicates mean fold change of gene expression in each time points. Red and green colors represent increased and decreased expression respectively. B–D) Quantitative RT-PCR analysis of *pax6a*, *shhb* and *dbx2* respectively showing fold change and differential pattern of expression in various time points after injury and compared with the temporal expression patterns of microarray data. Error bars represent SEM, n = 3, p<0.01. E–F) Quantitative analysis of PAX6 and SHH expression by ELISA in uninjured and injured cord at different time points. Error bars represent SEM, n = 5, p<0.001. G) Represents known genes related to dorso-ventral patterning in CNS that are differentially expressed at different time points after SCI. The color chart indicates mean fold change of gene expression in each time points. Red and green colors represent increased and decreased expression respectively. H–K) In situ hybridization of *dbx2* in uninjured (H), 3 dpi (I) and 10 dpi (J) cord section shows presence of *dbx2* transcripts in few neurons (black arrowheads) in uninjured cord but complete absence of *dbx2* transcripts in 3 dpi cord and reappearance of *dbx2* transcripts in many neurons in 10 dpi cord (black arrowheads). Significantly, higher numbers of *dbx2* positive cells are present in the ventral side of the 10 dpi cord compared to uninjured cord. Yellow arrows indicate neurons of ventral progenitor domain. Section in K represents sense control of *dbx2* in a 10 dpi cord. The mark ‘cc’ denotes central canal of the cord. ‘D’ and ‘V’ indicates dorsal and ventral side of the cord respectively. L) Representative transverse section of 10 dpi cord stained with HuC/D and DAPI showing the distribution of regenerated neurons in the dorso-ventral axis of the cord. Yellow arrows indicate neurons of ventral progenitor domain. M) A schematic diagram of various progenitor domains in dorso-ventral axis of spinal cord on the basis of spatial distribution and gene expression, adapted from Wilson and Maden [81]. ‘D’ and ‘V’ indicates dorsal and ventral side of the cord respectively. RP = Roof plate, FP = Floor plate, NC = Notochord, DP = Dorsal progenitor, VP = Ventral progenitor. Scale bar = 50 µm.

Regeneration is a complex process of rebuilding diverse tissue that involves patterning process and the mechanism substantially represents recapitulation of developmental pattern formation. The repatterning event has been studied in amphibian limb and spinal cord [74]–[77]. We have identified 84 pattern-forming genes that are differentially regulated (Fig. 7; Figure S6 and Table S13). Based on the temporal expression pattern we have separated 84 genes in 4 different groups. Highest numbers of genes (63 genes) are upregulated in 3 dpi, 10 dpi and 15 dpi cord that are downregulated in uninjured cord. Another category of genes (8) is upregulated in 1 dpi cord and gradually downregulated in later time points. All these genes are also down regulated in uninjured cord. The third and fourth groups, which include eight and five genes respectively, are upregulated in 7 dpi and 10/15 dpi cord. Genes belonging to these two groups are also upregulated in uninjured control unlike the first two categories (Figure S6 and Table S13).

Based on the studies on different species, combinatorial expression of different genes involved in dorso-ventral patterning of spinal cord have been identified [78]–[81]. Most importantly gradient of Shh induces five distinct classes of ventral neurons namely V_3_, V_MN_, V_2_, V_1_, V_0_ from neural progenitors [78], [80] and ventral cell types are determined by expression of different transcription factors. A gradient of SHH-N establishes a dorso-ventral domain by modulating expression of transcription factors of class-I and class-II proteins. SHH gradient is ventrally high and dorsally low, it represses class-I proteins creating an opposite gradient of *pax6*, *pax7*, *dbx1*, *dbx2*, *irx*. Similarly same SHH gradient activates class-II proteins namely nkx6.1 and nkx2.2 [79], [80]. Our present array analysis data indicates that upregulation of *shh* during regeneration probably creating gradient similar to development. Most of the genes involved in specification of different neuronal subtypes in ventral cord (Fig. 7G; Figure S6 and Table S13). For example *dbx2*, *irx3* and *pax6*, all involved in V_0_ and V_1_ neuronal patterning, are upregulated in regenerating cord similar to developing zebrafish spinal cord [82]. Similarly, *olig2*, *pax6*, *hlxb9* and *isl2*, involved in motor neuron progenitor domain, are also upregulated in regenerating cord. The neuronal populations identified in the dorsal domain is specified by expression of Mash, Math, Neurogenin and LIM homeobox proteins in developing vertebrate cord [81]. In regenerating cord we also observed upregulation of *neurog1*, *lim1* and *lim3b* (Fig. 7). We have validated three genes *dbx2*, *pax6a* and *shhb* by ELISA (Fig. 7E–F), qRT-PCR (Fig. 7B–D) and in situ hybridization analysis (Fig. 7H–K). Our qRT-PCR analysis revealed highest upregulation of all three genes in 10 dpi cord and significantly down regulated in control (Fig. 7B–D) and it suggests involvement of these genes in late events like repatterning of spinal cord. ELISA results of PAX6 and SHH confirms the expression pattern observed in array hybridization and qRT-PCR. *Dbx2* is another transcription factor involved in patterning of ventral spinal cord and specification of V_0_ and V_1_ domain interneurons during development [81]. Our array analysis and qRT-PCR data showed up-regulation of *dbx2* in 10 dpi and 15 dpi cord (fold change 8 and 6 respectively) and down regulation in other injured time points and uninjured control (Fig. 7D). In situ hybridization data reveals presence of few *dbx2* positive cells in the grey matter of uninjured cord and whereas 3 dpi cord showed complete absence of *dbx2* positive cells. The numbers of *dbx2* positive cells have increased significantly in 10 dpi cord compared to uninjured cord (Fig. 7H and J). *Dbx2* positive cells can be localized in the grey matter more ventrally, suggesting its possible role in respecifying ventral neuron during regeneration.

We have identified 52 genes, which are thought to be related to A–P patterning during CNS development in different species [83]–[85]. These genes are again differentially regulated at different time points after SCI. Based on the temporal expression pattern we observed three different groups. Majority of the genes (38 genes) showed upregulation in 3 dpi, 10 dpi and 15 dpi cord (21 genes are showing highest expression in 10 dpi and 15 dpi) but downregulated in uninjured control. Another set of genes showed upregulation both in uninjured control and 7 dpi cord but down regulated in other time points (Fig. 7A; Figure S6 and Table S13). A high number of genes (approximately 24) implicated in conferring A–P polarity of CNS belongs to four *hox* clusters. In regenerating zebrafish cord we observed six genes, viz. *hoxb7a*, *hoxb8a*, *hoxc8a*, *hoxd10a*, *hoxa11b*, *hoxd11a* which are all upregulated in 3 dpi, 10 dpi and 15 dpi cord. Upregulation of above mentioned *hox* genes in injured cord at later time points might suggest that they are probably involved in respecification of caudal spinal cord during regeneration. Furthermore, other patterning genes like *cdx1a* and *gdf11* are also upregulated in regenerating 3 dpi cord which are known to be involved in caudal spinal cord patterning during development [86], [87]. Thus, different *hox* genes with overlapping rostro-caudal domain of expression in the developing cord establish different region along the A–P axis [88], [89]. We inflicted injury at approximately 15^th^–16^th^ vertebrae level of adult spinal cord that is more caudal and any repatterning during regeneration would require respecification of spinal cord by combinatorial expression of relevant *hox* codes. More experimental validations involving different *hox* genes family members need to be done to corroborate our expression analysis.

#### Differentially expressed genes involved in axonal regrowth and guidance

Time course analysis of regeneration demonstrated axonal regrowth and substantial functional recovery after SCI in zebrafish [4], [6], [10]. We can see reconnected axons at 15 dpi cord although molecular events related to initiation of axonal regrowth may start early [6]. In the present study, we have identified many genes involved in axonogenesis and axonal guidance. There are at least 80 genes involved in axonal regrowth and guidance that also include many transcription factors (Figure S7 and Table S14). Based on temporal expression patterns, we have found 3 different groups. Group-I consists of 11 genes and they are upregulated both in uninjured control and 7 dpi cord. Group-II consists of 13 genes and they are particularly downregulated in control but upregulated in 1 dpi cord and gradually downregulated in later injury time points. In Group-III, highest number (56) of genes showed upregulation in 3 dpi, 10 dpi and 15 dpi cord but downregulated in uninjured control. It is important to note that IPA analysis also showed high enrichment of axonal guidance signaling genes in all three above mentioned time points (Fig. 2; Figure S2 and Table S3). Similar to developing nervous system there is also a complex mechanism of axonal pathfinding during axonal regrowth that include different axonal guidance molecules with attractive, repulsive and adhesive properties [90]. For example molecules like *robo1*, *robo2*, *slit1b*, *slit3*, *sema3ab* and *sema3h* all are with repellent properties and are all upregulated in 3 dpi, 10/15 dpi cord. Axonal attractant molecules like *netrin1a*, *netrin1b*, *plexina4*, are also upregulated in 3 dpi and 10 dpi cord. Cell adhesion molecules like *ncam* show slightly different expression pattern, as they are upregulated in 3 dpi and 10/15 dpi cord. Ephrins and receptor tyrosine kinases are known to be involved in organization of axons in different topographic planes during development of CNS [91], [92]. *Efnb1* and *efnb3* are upregulated in 3 dpi and 10 dpi cord whereas *rtk6* are upregulated in 1 dpi and 3 dpi cord but all remained downregulated in uninjured control. In developing zebrafish, motor guidance and target recognition molecules include CAMs [85], *semaphorins* [93], [94], *netrins* [93], robo, *slit* [95], *ephrin* and *rtk* [91]. Another gene *atf-3* is also highly upregulated in 1 dpi, 3 dpi and 10 dpi cord and may be involved in axonal regrowth as indicated in various reports [66], [96]. Induction of *atf-3* in both PNS injury and optic nerve injury is implicated in creation of permissive environment for axonal regeneration. Interestingly in zebrafish SCI, there is also creation of PNS like environment in CNS. As many as six genes (like *atf3*, *fgf2*, *mmp-9*, *stat3*, *socs3* and *sox11*), which are related to PNS regeneration, are upregulated after injury. Similar to the previous studies on axonal regeneration in zebrafish CNS [5], [97] *contactin1b* and *gefiltin* are also upregulated in either 10/15 day after injury. All these molecules are upregulated in regenerating cord during axonogenesis suggesting similar mechanism of axonal growth and guidance are probably being employed in directing axonal regrowth. In adult, functional analysis of identified genes during axonogenesis would provide some clue to understand regrowth of axonal tracts in regenerating cord.

### Expression of genes in signaling pathways during spinal cord regeneration

**Figure 8:**
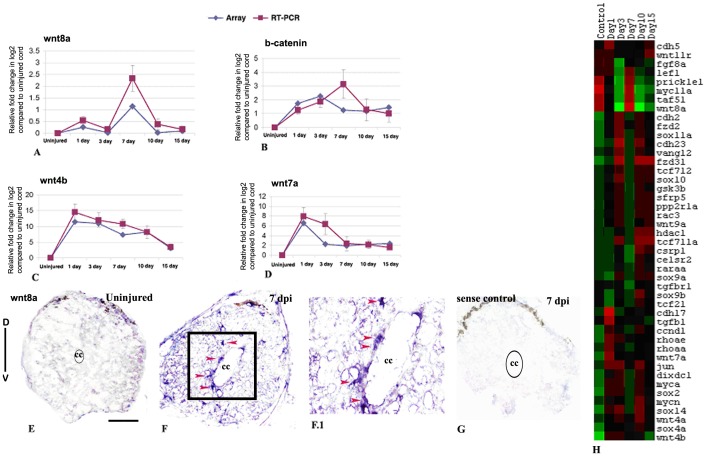
Differentially expressed genes involved in Wnt signaling pathway in zebrafish spinal cord after injury and validation of *wnt8a*, *wnt4b*, *wnt7a* and *b- catenin*: A–D) Quantitative RT-PCR analysis of *wnt8a*, *b-catenin*, *wnt4b* and *wnt7a* respectively showing fold change (red graph) and temporal expression pattern after injury. Pattern of expression was compared with microarray data (blue graph). Error bars represent SEM, n = 3, p<0.01. E–F) In situ hybridization with *wnt8a* anti-sense probe showed absence and presence of mRNA transcripts in uninjured and 7 dpi cord respectively. F.1) Higher magnification of the boxed area in section F shows *wnt8a* expression in ependymal cells (red arrowheads). G) In situ hybridization of 7 dpi section with *wnt8a*sense probe as control. ‘D’ and ‘V’ indicates dorsal and ventral side of the cord respectively. The mark ‘cc’ denotes central canal of the cord. H) Represents temporal expression pattern of genes after SCI related to Wnt signaling pathway. The color chart indicates mean fold change of gene expression in each time points. Red and green colors represent increased and decreased expression respectively. Scale bar = 50 µm.

We have analyzed expression profiles of different members of Wnt, BMP/TGF beta and JAK-STAT pathway and validated expression of few genes from the respective pathways (Fig. 3, 4, 8; Figure S8 and Table S15). Other signaling pathways such as Hedgehog, Notch and FGF also appear to play critical role in regeneration (Figure S8 and Table S15).

#### Wnt signaling

Many members of Wnt signaling pathway are differentially regulated at different time points suggesting their diverse role in cellular events of regeneration (Fig. 8H; Figure S8 and Table S15). Several Wnt members like *wnt4a*, *wnt4b*, *wnt7a*, *wnt8a*, *wnt9a*, *wnt11* and *β-catenin* are upregulated at different time points after injury. Other members of Wnt signaling pathway represent Frizzled like (*fzd2*, *fzd3i* and *celsr2*; zebrafish homolog of mouse *flamingo*) which are mostly upregulated in 3 dpi and 10 dpi cord. Both *wnt4a* and *wnt9a* are upregulated in 3 dpi and either in 10 and or 15 dpi cord and downregulated in other time points of regeneration. On the other hand, *wnt8a* showed upregulation in control and 7 dpi cord and down regulated in other time points. Regulators of Wnt signaling genes like *dixdc1*, *gsk3b*, *tcf711* and *tcf712* are all differentially upregulated in 3 dpi, 10 dpi and 15 dpi cord and down regulated in 7 dpi cord. There are different inducible targets of different Wnt, β-catenin or Frizzled, which include *myca*, *mycn*, *myc11a*, *jun*, *ccnd1*, retinoic acid receptors and many members of *sox* family genes. Role of different Wnts like *wnt3a*, *wnt5a*, *wnt5b*, *wnt7a*, *wnt8a*, *wnt10a* are well documented in different regenerating tissues [56], [98]–[101]. In regenerating fin, over expression of *wnt8a* caused activation of *β-catenin* and which leads to increased proliferative response and accelerated regeneration [100]. We have validated array expression data of *wnt8a* and *β-catenin* by qRT-PCR and in situ hybridization experiments (Fig. 8). The qRT-PCR analysis shows both *wnt8a* and *β-catenin* are upregulated in 7 dpi cord, where we see highest rate of proliferation (Fig. 8A–B). In situ hybridization analysis with *wnt8a* transcript showed high expression in 7 dpi cord and absent in uninjured and 3 dpi cord. Many cells with *wnt8a* mRNA transcript are present around the central canal and are ependymal cells (Fig. 8F) which are thought to be precursors [12]. These data confirm our previous observation where we have showed the presence of proliferating BrdU^+^ precursors in ependyma [6]. Few *wnt8a* expressing cells are also present in white matter of 7 dpi cord (Fig. 8F and F.1). The spatio-temporal distribution of *wnt8a* transcript in regenerating cord suggests that it is upregulated predominantly in ependymal cells, which actively proliferates following injury. Another member of Wnt family *wnt7a* is upregulated in wound epidermis, cartilage, spinal cord and muscle in regenerating tail in urodele [102]. Both *wnt7a* and *wnt4b* are highly upregulated in regenerating cord in early time points and validated by qRT-PCR (Fig. 8C–D. Apart from the positive regulators there are many negative regulators in Wnt signaling pathway like *dixdc1*, *gsk3*, *sfrp1*, *sfrp1b* and *sfrp5* suggesting both presence of pro and negative Wnt signaling during spinal cord regeneration.

#### BMP/TGF beta signaling

Members of BMP/TGF beta signaling pathways are differentially up or down regulated during the different events of regeneration (Figure S8A and Table S15). This list contains both TGFβ signaling genes and their target genes. We observe different group of genes based on temporal expression pattern. Among members of TGFβ signaling family, temporal expression pattern of *tgfβ1* gene is distinctly different being upregulated immediately after injury (1 dpi cord) and down regulated in subsequent time periods. This gene is associated with inflammatory process early during regeneration phase as discussed previously in section 3A. Members of BMPs and growth and differentiation factor 11 (Gdf11) are known to control neurogenesis in olfactory neuroepithelia and help in maintenance of progenitor population by replacing dying neurons [103], [104]. We have also found *bmp5* and *gdf11* both are upregulated in 3 dpi and 10 dpi cord and these genes are known to play key role in this pathway.

#### JAK-STAT signaling

Three members of JAK-STAT pathway like stat1b, stat5.2 and stat3 are upregulated during regeneration of zebrafish spinal cord (Figure S8B and Table S15). Stat5.2 and stat1b are both upregulated at 3 dpi and 10 dpi cord and down regulated in uninjured control and other injury time points. Stat3 showed different temporal expression pattern than others and uprgulated in 1 dpi and 3 dpi cord. Similarly, stat3 interacting genes like rtk6 and target genes like socs3a and socs3b, mapk2l and ptpn6 all are upregulated in 1 dpi and 3 dpi cords. Stat3 expression has been validated and described previously (Fig. 3), which is an important regulator of inflammation and may promote cell survival and proliferation. The temporal expression of stat3 and its downstream targets like socs3a and socs3b are similar and may indicate the immunomodulatory role of socs3 by regulating JAK-STAT signaling as suggested earlier [105].

### Identification of conserved genes differentially expressed during spinal cord, retina, fin and heart regeneration in zebrafish

**Figure 9:**
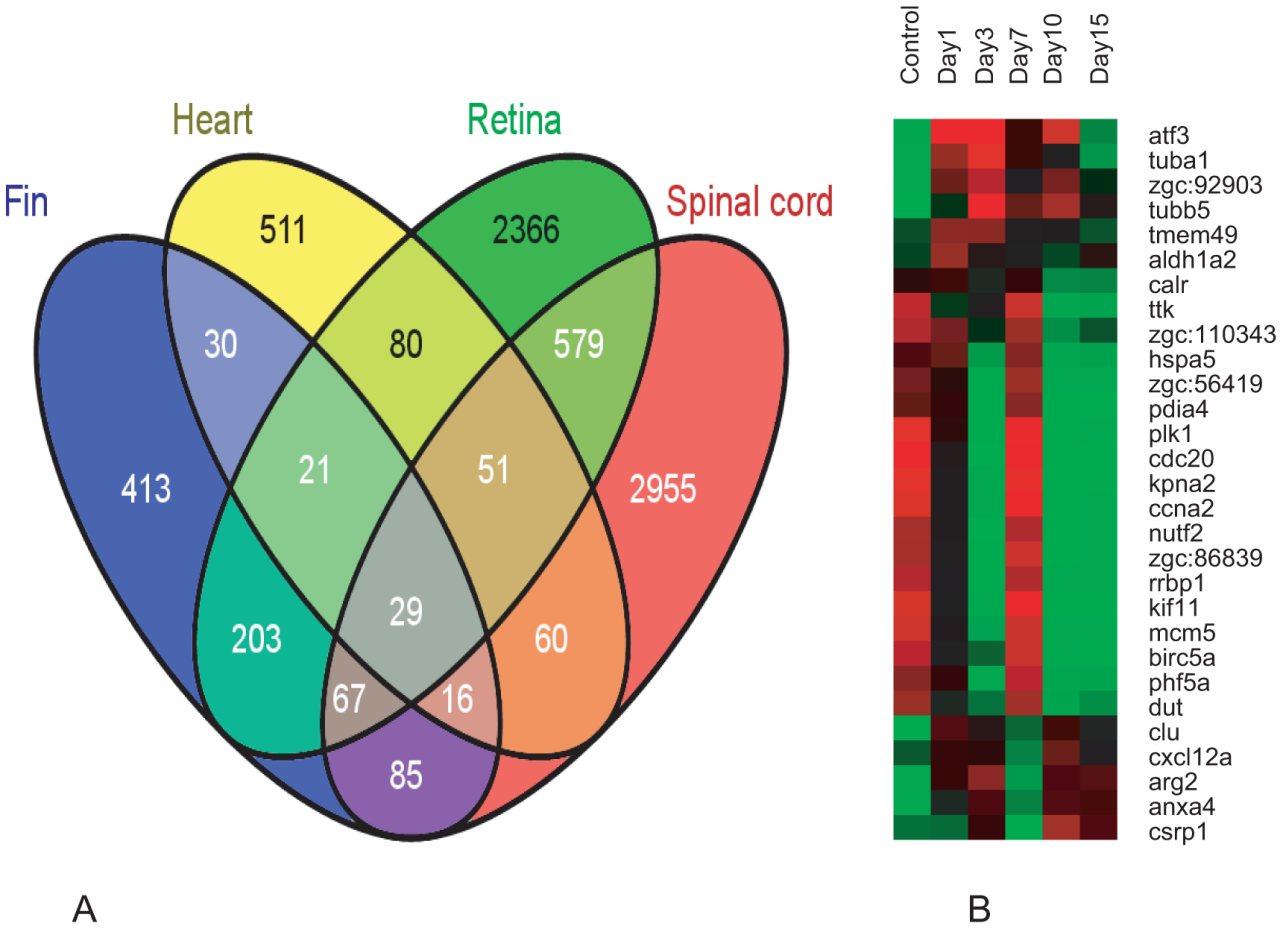
Common genes differentially expressed in spinal cord, fin, retina and heart during regeneration in zebrafish. A) Vein diagram illustrates number of genes that are expressed (uniquely as well as shared) during regeneration of the four different tissues like fin, heart, retina and spinal cord. B) Differential expression pattern of the 29 common genes in uninjured and injured zebrafish spinal cord with different time points. The color chart indicates mean fold change of gene expression in each time points. Red and green color represents increased and decreased expression respectively.

To identify the conserved genes involved in regeneration, we compared the gene identified in regenerating spinal cord with the set of genes identified in other regenerating tissues like heart, fin and retina. Among the 3842 genes that are differentially expressed during spinal cord regeneration, 156, 297 and 835 genes are commonly expressed during heart, fin and retinal regeneration respectively (Fig. 9A; Table S16). Both spinal cord and retina regeneration involve related common process like proliferation and neurogenesis and they shared maximum number of common genes during the regeneration. It is evident from the figure 9A that these tissues share a significant number of genes among themselves whether they are compared in twos or threes. When we consider all the four tissues at a time, we find they share 29 common genes (Fig. 9B). However there are also 2952, 2362, 427 and 511 genes that are specific to regenerating spinal cord, retina, fin and heart respectively (Fig. 9A).

There are five known genes *plk1* (polo-kinase 1), *ttk1*/*mps1* (monopolar spindle 1, a kinase required for mitotic check point regulation), *cdc20*, *ccna2* and *atf-3* commonly expressed in different regenerating organs like fin, heart, retina and spinal cord (Fig. 9B; Table S16). Among these genes, expression of *mps1* has been reported to be present in fin blastema [3], regenerating heart [15] and retina [5] during proliferation phase. We report low and high expression of mps1 in uninjured and 7 dpi cord respectively. Similarly, M-phase regulators like *plk1*, *cdc20*, and *ccna2* are also upregulated in 7 dpi cord. These data suggest that there is a common mechanism regulating cell proliferation in all these regenerating organs and all the three M-phase regulators are tightly controlled during proliferation. *Atf3* is another common gene which is highly upregulated in regenerating cord compared to normal uninjured cord.

*Hdac1* is yet another common cell cycle regulator known to repress expression of *ccnd1* and *ccne2* [106] and expressed in regenerating CNS like retina [107] and in developing CNS [108]. Temporal expression pattern of *hdac1* shows two different upregulated peaks in 3 dpi and 10/15 dpi cord and may be related to gradual repression of *ccnd1* from 3 dpi to 15 dpi cord. Furthermore, second peak of *hdac1* expression in 10 dpi and 15 dpi cords is suggestive of its role in differentiation of neurons and glia as observed in zebrafish retina [106], [107].

### Many genes involved with one-carbon folate metabolism, N-glycan biosynthesis and ion transport were differentially expressed during spinal cord regeneration

Based on the IPA analysis we have identified and clustered at least 44 genes which are involved in ion transport and are also differentially expressed during regeneration (Figure S9 and Table S17). This particular group includes genes of sodium, potassium and calcium channel proteins; glutamate, glycine and purinergic receptors and other associated ion transport proteins.

The IPA analysis also identified two novel signaling pathways like one-carbon folate metabolism and N-glycan biosynthesis from the array data and the genes involved in these signaling pathways have been identified (Figure S9 andTable S17). All the five genes (*tyms*, *dhfr*, *shmt1*, *atic* and *amt*) involved with one-carbon folate metabolism are upregulated in uninjured zebrafish cord and 7 dpi cord. Following transection injury to the rat spinal cord, increased expression of folate receptor 1 (FOLR1) promotes methylation of spinal cord DNA [109], [110]. Two genes, *tyms* and *dhfr* are also involved in de novo purine-pyrimidine biosynthesis. Temporal expression pattern of these 5 genes further corroborates with our finding in IPA analysis that enrichment of purine-pyrimidine metabolism pathway genes are highest (63%) at 7 dpi cord.

Among the genes related to N-glycan biosynthesis pathway, four genes are upregulated in both uninjured and 7 dpi cord. Only *b4galt1* is upregulated in 3 dpi and 10/15 dpi cord and remained downregulated in uninjured control and other injured time points. All the five genes (*b4galt1*, *st6gal1*, *alg3*, *alg1* and *mgat3*) in this group are enzymes related to glycosylation of proteins.

### Identification and validation of some unannotated genes involved in spinal cord regeneration

**Figure 10:**
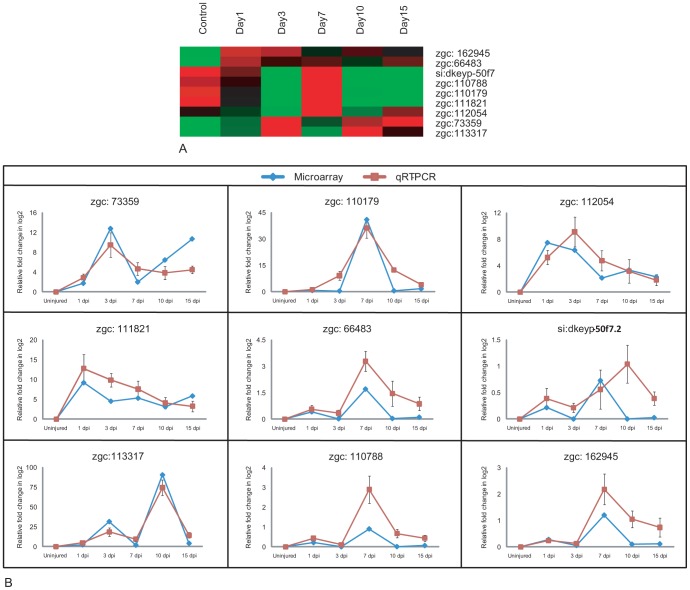
Validation of unannotated genes differentially expressed in array hybridization during regeneration of zebrafish spinal cord. A) Cluster of nine differentially expressed unannotated genes. Each horizontal line indicates the expression pattern of each gene and the vertical columns indicate the uninjured control and time points after SCI. The color chart indicates mean fold change of gene expression in each time points. Red and green colors represent increased and decreased expression respectively. B) Comparative gene expression analysis by microarray (Blue) and qRT-PCR (Red) of nine unannotated genes at different injury time points. Error bars represent SEM, n = 3, p<0.05.

We have identified 91 genes that are totally uncharacterized and unannotated from the list of differentially expressed genes. Although these genes have unique Unigene ID their functions are not properly annotated; few of them have unannotated/validated human homolog. As of now, it appears that these are fish specific genes that are involved in regeneration process. Identification of human homolog for these fish specific genes will give new target genes, which may be crucial in regeneration process. Among the 91 uncharacterized genes, we selected 9 genes that show a high fold change and are distributed among the 7 clusters represented in figure 1 (Fig. 10; Table S18). Expression pattern of these genes are represented in treeview (Fig. 10A) and are validated by qRT-PCR (Fig. 10B). We have BLAST searched the proteins of these nine genes to find their putative homologues in human and assigned functions to them based on these homologues. The expression patterns and the human homologous functions of the genes are described below. Among these nine genes, four are upregulated both in uninjured and 7 dpi cord which are zgc:66483 (zinc finger protein 389), si:dkeyp-50f7.2 (ZPA domain containing protein), zgc:162945 (Putative nuclease Harbi1) and zgc:110788 (Vesicle transport protein Sft2B). zgc:110179 (Ras signaling related protein) is upregulated in 7 dpi and 15 dpi cord. zgc:111821 (Spp1 protein or Osteopontin) and zgc:112054 (cAMP responsive element) are upregulated in 1 dpi and 3 dpi cord. Other two genes are upregulated in 3 dpi and 10/15 dpi cord which are zgc:73359 (Retinal rod rhodopsin sensitive cGMP) and zgc:113317 (Amine sulfotransferase) (Fig. 10).

**Figure 11:**
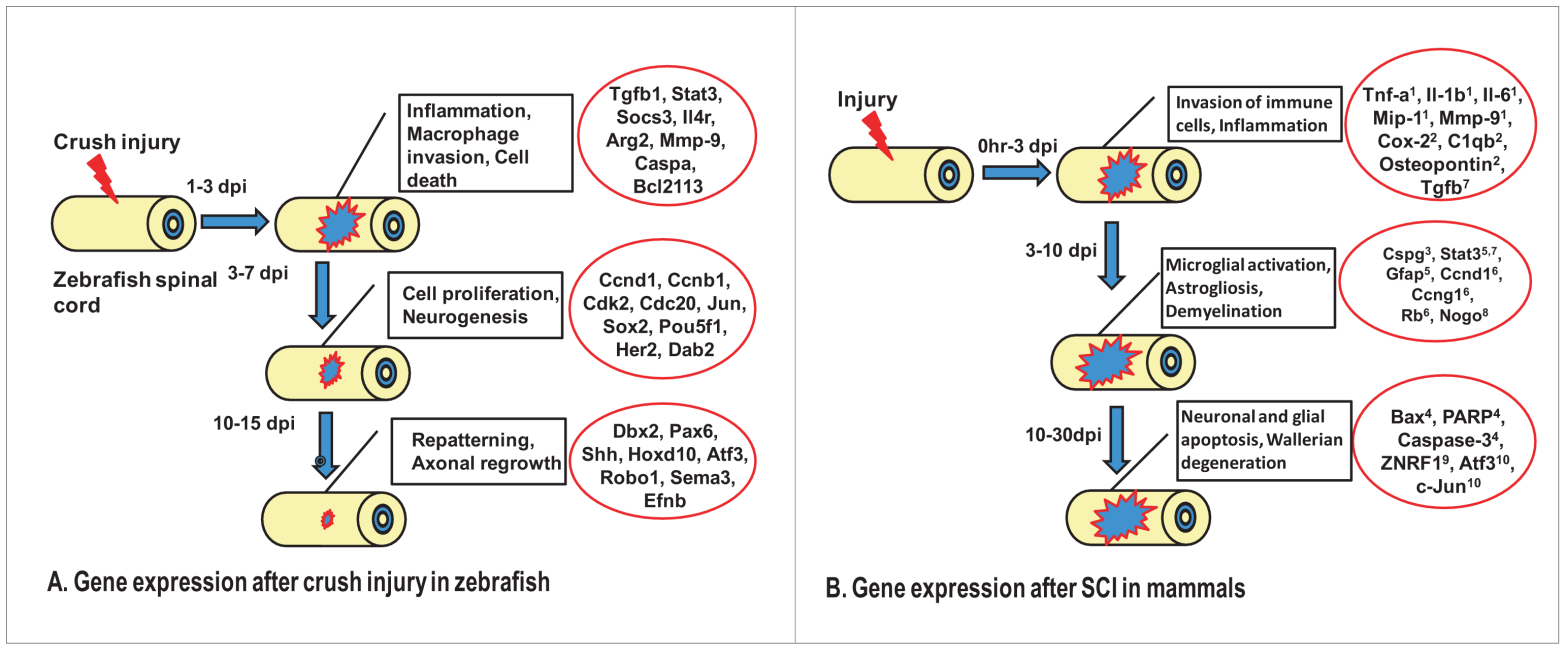
Comparative analysis of cellular events and upregulation of genes following SCI in zebrafish and mammals. A) Schematic representation of different events and underlying expression of some important genes during zebrafish spinal cord regeneration. B) Schematic representation of different events and corresponding upregulation of genes in mammalian spinal cord after inflicting different modes of injury. The expressions of gene(s) are compiled based on previous evidences, which are known to regulate different cellular events. (1. [120]; 2. [121]; 3. [122]; 4. [123]; 5. [111]; 6. [112]; 7. [124]; 8. [125]; 9. [126]; 10. [127].).

We have used zebrafish as a regeneration competent model and combined the transcriptome profiling to uncover the molecular mechanisms underlying spinal cord regeneration. Our analysis is based on different cellular events occurring during regeneration and thus allowed us to specify molecular basis of different cellular events that are different from one another. A schematic diagram represents the different cellular events and upregulation of some important genes expression (Fig. 11). The diagram also highlights the similarity and dissimilarity of the events between zebrafish and mammalian SCI thus providing more insight on how to approach for possible future therapeutic strategy. In summary, some of the key events analyzed are inflammatory response, cell proliferation & neurogenesis and axonal growth and inhibition. Early inflammatory response was induced after injury both in zebrafish and in mammals, although nature of cells involved in inducing inflammatory response varies. Some of the genes (*tgfb1*, *stat3* and *casp3*) are conserved in responding to injury between the two species. In mammalian SCI, astrogliosis and microglial activation is key to regeneration failure. In fish CNS, microglial activation is rapid but transient and proved to be beneficiary [9]. One of the expression markers of glia, GFAP is downregulated after SCI [6] whereas GFAP upregulation has been observed in mammalian SCI [111]. Proliferative response is high following injury in zebrafish cord. Expression of different cell cycle regulators and transcription factors points towards their contribution in neurogenesis. In comparison, proliferative response in mammals predominantly targets astrocytes and inflammatory cells [112]. Failure of axonal regeneration in mammal is controlled by absence of permissive niche, apoptosis, demyelination followed by accumulation of myelin debris and Wallerian degeneration [113], [114]. We observed limited apoptosis, rapid debri clearance, and absence of Nogo (Our unpublished observation) and generation of permissive niche. Expression of appropriate genes responsible for axonal regrowth has also been documented in zebrafish cord.

## Conclusions

The present study has identified 3,842 differentially expressed genes during spinal cord regeneration in zebrafish by using high-density oligonucleotide based microarray. Events specific cluster analysis further identified many important genes involved in controlling different processes in regeneration like inflammation, cell death, cell migration, cell proliferation and neurogenesis followed by axonogenesis and repatterning of the regenerating spinal cord tissue. Comprehensive event specific analysis during spinal cord regeneration is a key to define therapeutic strategy to be adapted in higher vertebrates. Possibility of using a combinatorial approach may include targeting of genes a) to control secondary degenerative response after injury, b) to induce controlled cell proliferation, c) to induce selective neurogenesis and axonogenesis and d) for creation of permissive niche. All the cellular events mentioned here are to be taken care of simultaneously in order to deliver appropriate therapy after SCI in mammals. There are also lessons to be learnt about the evolutionarily common genetic program operating in different organs and information on tissue specific gene expression that could be used selectively for regeneration of specific organs.

## Materials and Methods

### Zebrafish husbandry and surgical procedure to inflict spinal cord injury

Zebrafish were obtained (∼3–4 cm) either from local pet shop or bred in our animal house facility. Fish were kept in separate groups of 10 in the aquatic system maintained at 28°C on a 14 hr light/10 hr dark cycle. Fish were anaesthetized for 5 minutes in 0.02% tricaine (MS222; Sigma, USA) before giving SCI. A longitudinal lesion was made at the side of the fish to expose vertebral column at the level of dorsal fin, which corresponds to 15/16th vertebrae. The spinal cord has been injured by crushing dorso-ventrally for 1 sec with a number 5 Dumont forceps at the same level (Figure S10). Later the wound were sealed by placing a suture. Both spinal cord injured and sham operated fish were allowed to regenerate and the progress of regeneration was observed after 1, 3, 7, 10 and 15 days of injury. During the process of regeneration, operated fish were incubated in normal aquarium at 28°C . This study was carried out in accordance with the recommendation in the guidelines provided by CPCSEA (Committee for the Purpose of Control and Supervision of Experiments on Animals, Ministry of Environments and Forests, Government of India). The protocol (Part B), which includes all the details of surgical processes inflicted and anesthesia requirement for injuring and sacrificing animals, was approved by the Institutional Animal Ethics committee, Department of Biophysics, Molecular Biology and Bioinformatics, University of Calcutta under the registration number with CPCSEA (CPCSEA/ORG/CH/Reg No. 925/295).

### Tissue collection and RNA extraction for microarray analysis

Control fishes and spinal cord injured fishes were anesthetized deeply for 5 minutes in 0.1% tricaine (MS222; Sigma, USA) and approximately 1 mm length of spinal cord both rostrally and caudally from injury epicenter were dissected out from 50–60 fishes in each batch and pooled for RNA extraction. RNA was extracted from the control and regenerating spinal cords of the experimental fishes using Trizol (Invitrogen,15596018) and quality of RNA was checked on Agilent 2100 Bioanalyzer (Agilent RNA 6000 Nano Assay). RNA was prepared from a minimum of 3 biological replicates. For each biological replicate, spinal cord from about, 50–60 fishes were pooled in the control and in the regenerating samples.

### Oligonucleotide probe design and microarray design

Microarray was performed using Agilent platform. Zebrafish microarray was custom designed for gene-expression, containing ∼44,000 probes (60 mer long; including positive and negative controls designed by Agilent and beta-actin controls). All procedures were carried out using standard recommended protocols from Agilent. The microarrays were performed following Agilent's One-Color Microarray-Based Gene Expression Analysis (Quick Amp Labeling) protocol (Version 5.7, March 2008) and RNA Spike-In-One Color. We used about 400 ng total RNA as the starting material for amplification and labelling. After cDNA and cRNA synthesis, the Cy3 labeled cRNA samples were column purified, and checked for yield and those passed the QC (1.65 µg and specific activity >9.0 pmol Cy3 per µg cRNA) were used for hybridization to the arrays. Arrays were hybridized at 65°C for 17 hours in a slow rotating hybridization chamber. The slides were washed as described in Agilent's protocol and scanned on Agilent's DNA Microarray Scanner. The scanned images were analyzed using Agilent Feature Extraction Software (v10.5.1.1). Feature extracted data were analyzed using the tools in GeneSpring. Full microarray data were deposited in GenBank, GEO accession number is GSE39295 and it is available in following link http://www.ncbi.nlm.nih.gov/geo/query/acc.cgi?acc=GSE39295.

### Statistical analysis and selection of differentially expressed genes

Statistically significant gene expressions were identified using Significance Analysis of Microarrays (SAM 3.05) for each successive time point [115]. Stringent values of fold-change and other thresholds are taken into account while identifying differentially expressing genes. The threshold values we selected are stringent, where the fold change is always greater than or equal to 2 and the q-value is always less than 0.8. The predicted false discovery rate (FDR) never exceeded 0.05%. FDR indicates the expected proportion of false positives among the declared significant results. A final set of differentially expressed genes was then obtained after removing all the repetitions. A PERL script is used to filter out the annotated differentially expressed genes from the un-annotated genes.

### Annotation of genes

Annotations of genes were done using the “Unigene & Gene Ontology Annotation Tool” available at GIS site (link: http://123.136.65.67/). We have mapped the Unigene IDs against this database and retrieved the zebrafish UniGene description (UniGene build 119), Entrez Gene ID, Official Gene symbol, GO term and Human homologues information (Unigene ID and Descriptions).

### Identification of gene expression pattern

The expression values of the technical replicates of each gene was then averaged and then used for further analyses. Next, we have identified the peaks for each gene (i.e. time point where the expression value of the gene is highest in the array). Genes with similar functions may show similar expression pattern, at least similar time of highest activity. Therefore, we have grouped the genes with similar peak expression time point, performed the cluster analysis using Gene Cluster 3.0 [116], viewed using the software Java TreeView [117], and then carried out the functional enrichment analysis for each cluster using the GO annotation.

### Functional event wise clustering of differentially expressed genes by IPA analysis

The differentially expressed genes were subjected to Ingenuity Pathways Analysis (IPA) to identify the enrichment of genes in specific functional groups and pathways (IPA, Version 4, Ingenuity Systems, http://www.ingenuity.com). IPA can accept zebrafish as well as Human UniGene IDs as input for data analysis. We used IPA to find functional and pathway enrichment in the gene sets that are differentially up and down regulated at different time points of regeneration. The clusters resulting after k-means clustering were also analyzed using IPA.

### Microarray validation by using quantitative real-Time RT-PCR (qRT-PCR)

The total RNA was extracted from both normal and injured spinal cord by RNaqeous-4PCR kit (Ambion, USA) and cDNA was synthesized using 2 steps RT-PCR (Retroscript, Ambion, USA) with equal amounts of total RNA and by using specific oligo dT primers. Real-Time quantitative RT-PCR using relative quantitation by the comparative C_T_ method was used to determine mRNA expression. 3 µl of cDNA was subjected to Real-Time quantitative RT-PCR using the Real Time PCR (ABI-7500, USA) with SYBR Green as a fluorescent reporter using the qPCR master-mix plus for SYBR Assay I Low ROX (Eurogentec, USA). The specific gene primers (zebrafish *sox2*, *stat3*, *tgfβ1*, *dbx2*, *wnt8a*, *cdk2*, *ccnb1*, *ccne*, *ccnd1*, *pax6a*, *shhb*, *wnt4b*, *wnt7a* and *β-catenin*) and the internal control gene *β-actin* (beta-actin) were amplified in separate reaction tubes. The details of all known gene primers used are tabulated in Table S19. From the microarray dataset, the genes that do not have UniGene annotation (unannotated genes) were also validated by qRT-PCR. The details of specific gene primers for each gene and the internal control gene *β-actin* (beta-actin) are listed in Table S20. Threshold cycle number (C_T_), of triplicate reactions, was determined using the ABI-7500 software and the mean C_T_ of triplicate reactions was determined. The levels of specific gene expression was normalized to beta-actin levels using the formula 2^−ΔΔC^T, where ΔΔC_T_  = ΔC_T_ (sample)−ΔC_T_ (calibrator) and ΔCT is the C_T_ of the housekeeping gene (beta-actin) subtracted from the C_T_ of the target genes. The calibrator used in our experiments is the uninjured spinal cord tissue and the samples are injured spinal cord tissue of same time points as used in microarray. The ΔC_T_ values are being inversely proportional to the mRNA expression of the samples. No primer -dimers were obtained for either the target genes or beta-actin as assessed by melt curve analysis. The specificity of the products was also confirmed by melt curve analysis. The PCR cycles in all cases were started with Taq activation at 94°C for 5 mins and followed by final extension of 72°C for 7 mins. All experimental data are expressed as Mean±SEM. The data obtained were compared using unpaired two-sided Student's *t*-test, Significance was set at *P*<0.05. Data were analyzed using Excel software.

### Immunohistochemistry

Spinal cord tissues were dissected out and fixed in 4% paraformaldehyde (Sigma, USA) for 8 hr or overnight at 4°C. Both injured and uninjured spinal cord tissues were embedded either in paraffin or in a mixture of PEG and hexadecanol (9∶1, Sigma) or in O.C.T compound (Leica, Germany) and sectioned at 5–7 micron. Immunostaining was performed as described previously [6] by using the following primary antibodies shown to specifically recognize fish, amphibian and human proteins: anti- HuC/D (1∶50, Molecular Probes, USA), anti- TGFβ (1∶100, Santa Cruz Biotech, USA), anti- STAT3 (1∶100, Santa Cruz, USA), anti- GFAP (1∶400, DAKO), anti- SOX2 (1∶200, Abcam, USA), anti- BrdU (1∶200, Sigma, USA), anti- Phospho-histone H3 (H3P, 1∶400, Cell Signaling Technology, USA). Tissue sections were briefly rehydrated in phosphate buffered saline (PBS) and given several washes in PBS with 0.1% Tween-20 or Triton X-100 (PBST). The sections were first incubated with blocking solution for 1 hr (5% goat/rabbit/donkey serum, 1% BSA in PBS) and then with primary antibody for either 1 hr at room temperature or overnight at 4°C. Antigen retrieval was done wherever appropriate by keeping the slides in 85°C water bath for 15 min either in sodium-citrate buffer (pH 6.0) or Tris buffer (pH 8.0) before incubation with the primary antibody. The following secondary antibodies were used: Rhodamine-conjugated goat anti-rabbit antibody (1∶100, Santa Cruz, USA), rhodamine-conjugated donkey anti-goat antibody (1∶100, Santa Cruz, USA), rhodamine-conjugated goat anti-mouse IgG antibody (1∶100, Jackson ImmunoResearch Laboratories, USA), FITC-conjugated goat anti-mouse (1∶50, Santa Cruz, USA) and FITC-conjugated goat anti-rabbit IgG antibody (1∶100, Jackson ImmunoResearch Laboratories, USA). Nuclei were counter-stained either with bisbenzimide H 33258 fluorochrome (Hoechst nuclear stain) or with DAPI (1∶1000, Sigma, USA). We injected 50 µl volume of BrdU (Bromo-deoxy- Uridine, Sigma-Aldrich, USA) at the concentration 2.5 mg/ml to the adult fish intra-peritoneally, 24 hr before the collection of tissues at different injury time points. Spinal cord tissue equivalent of approximately 1 mm length was collected from both injured and uninjured animals. Tissue samples from injured cord was collected in such a way that epicenter of the lesion is going approximately through middle of the cord and included normal part on the both sides of the injury. BrdU immunohistochemistry on uninjured and injured spinal cord was done according to Hui et al. [6]. Paraformaldehyde fixed paraffin sections were washed in PBST twice for 10 min, followed by incubation in 2N HCl (pH 2.0) for 30 min at 37°C. Sections were washed in PBS and blocked with 5% normal goat serum in 0.5% PBST for 1 hr at room temperature. The mouse monoclonal anti-BrdU primary antibody (1∶300, Sigma, USA) was applied for 2 hr at room temperature or overnight at 4°C. This was followed by washes in PBS and incubated with secondary anti-mouse FITC/TRITC conjugated antibody for 2 hr at room temperature. After washing in PBS, the sections were mounted with Aqua Vectashield (Vector labs, USA) and kept in the dark. During H3P and BrdU colocalization study, the quantification of cells was done from all optical images by using a Zeiss LSM 510 Meta (inverted) confocal microscope. We have used approx. 4 mm length of tissues both from uninjured (n = 4) and injured cords (n = 4), sectioned the whole cord and all the sections were stained with both H3P along with BrdU and counter-stained with DAPI. All quantification data are expressed as Mean±SEM. using Excel software. The data obtained were compared using unpaired two-sided Student's *t*-test, Significance was set at *P*<0.05.

### Enzyme linked immunosorbant assay (ELISA)

Analysis of TGFβ, PAX6 and SHH by using ELISA assay was done according to Dutta et al. [118]. Tissue extract of spinal cord were added to wells (65 µg protein/well) of the ELISA plate and incubated overnight at 4°C. After non-specific sites were blocked with blocking buffer (1% BSA in PBS), the wells were incubated with primary antibodies like anti- TGFβ (1∶100, Santa Cruz Biotech, USA), anti- PAX6 (1∶30, Developmental Studies Hybridoma Bank, USA), anti- SHH (1∶50, Developmental Studies Hybridoma Bank, USA) for 1 hr at 37°C, and washed thrice in washing buffer (0.5% BSA, 0.5% NP-40 in PBS). The HRP coupled anti- goat, anti- mouse, anti-rabbit antibodies (1∶1000 dilution) were used for incubating the plates at 37°C for 1 hr, then washed several times in washing buffer and substrate tetramethyl benzedine (TMB) was added in dark. The reaction was stopped by adding 1M H_2_SO_4_. The optical density was measured at 450 nm by using an ELISA reader (Bio Rad, USA). Data are expressed as Mean±SEM using Excel software. The data obtained were compared using unpaired two-sided Student's *t*-test, Significance was set at *P*<0.05.

### In situ hybridization on zebrafish tissue section

To obtain riboprobes for in situ hybridization pCRIITOPO vector (Invitrogen) containing *wnt8a* and *dbx2* cDNA clones were obtained from Genome Institute of Singapore, zebrafish genome resources. Both sense and antisense RNAprobes were generated using a digoxigenin (DIG) RNA labeling kit (Roche Diagnostics, Laval, Que'bec, Canada) following the manufacturer's instructions. The probes were used to detect *dbx2* and *wnt8a* mRNA in zebrafish tissue section. For tissue sections, both uninjured and injured cord were excised and quickly fixed in precooled 4% paraformaldehyde prepared with DEPC-PBS in RNAse free condition, cryo protected with 20% sucrose in PBS and frozen with OCT compound and subsequently stored at −80°C. Cryostat sections (10 µm) were cut and fixed to poly-L-lysine-coated slides and allowed to dry. In situ hybridizations were performed following the protocol of Braissant and Wahli [119], with the addition of a 5 minutes digestion with proteinase- K (5 µg/ml) diluted in 50 mM Tris HCl (pH 8, 5 mM EDTA) step before prehybridization. Briefly, after fixation in 4% paraformaldehyde, tissue sections were washed and prehybridized for 2 h at 58°C in 5× saline sodium citrate (SSC)/formamide (1∶1) containing 40 µg/ml salmon sperm DNA, 50 µg/ml yeast tRNA, 4 mM EDTA, 2.5% dextran sulfate). The sections were then incubated overnight at 58°C in SSC/formamide containing 1 µg/ml DIG-labeled *dbx2* sense or antisense RNA probe and at 60°C in SSC/formamide containing 1 µg/ml DIG-labeled *wnt8a* sense or antisense RNA probe. After hybridization, sections were washed in 2× SSC at room temperature (RT) and then 2× and 0.1× SSC at 65°C. After Blocking with in situ blocking reagent (Roche Diagnostics, Laval, Que'bec, Canada) at 37°C for 2 hrs, sections were washed again in 0.1× SSC and incubated with an anti-DIG antibody (Roche; 1∶5000 dilution) for overnight in 4°C. Color was developed using 5-bromo-4-chloro-3-indoyl-phosphate/4-nitro blue tetrazolium chloride (NBT-BCIP, SIGMA) for 2 to 12 hrs at room temperature (RT). Photographs were taken under light microscope using an Olympus microscope (model; BX 51) and a Leica Microsystem microscope with build camera (DFC 290).

## Supporting Information

Figure S1 **Pie charts showing the percentage of functionally enriched genes that are differentially expressed in the seven clusters.**
(JPG)

Figure S2 **Pie charts show the percentage of functionally enriched genes that are differentially expressed at different time points after SCI in zebrafish.**
(JPG)

Figure S3 **Pie charts showing the percentage of canonical pathway enriched genes that are differentially expressed at different time points after SCI in zebrafish.**
(JPG)

Figure S4 **Quantification of cells present in different cell cycle phases in uninjured and injured cord based on BrdU, H3P and DAPI colocalization study.** A) In uninjured cord only 2% and 1% of cells are in S-phase and M-phase respectively. B) In 3 dpi cord the percentage of S-phase cells have been increased significantly to 8% than uninjured cord and only 2% cells are in M-phase. C) In 7 dpi cord 12% of total populations are in S-phase and 5% of total populations are in M-phase.
(JPG)

Figure S5 **Dendrogram represents differential expression pattern of transcription factors involved in neurogenesis and neuronal specification during regeneration of zebrafish spinal cord.** Each horizontal line indicates the expression pattern of each gene and the vertical columns indicate the uninjured control and time points after SCI. The color chart indicates mean fold change of gene expression in each time points. Red and green color represents increased and decreased expression respectively.
(JPG)

Figure S6 **Differentially expressed genes related to pattern formation are represented in two dendograms in regenerating zebrafish spinal cord.** Each horizontal line indicates the expression pattern of each gene and the vertical columns indicate the uninjured control and time points after SCI. The color chart indicates mean fold change of gene expression in each time points. Red and green colors represent increased and decreased expression respectively.
(JPG)

Figure S7 **Differentially expressed genes related to axonogenesis and axonal guidance is represented in two different dendrograms (A and A1) in regenerating zebrafish spinal cord.** Dendrogram (B) represents differential expression pattern of transcription factors involved in axonogenesis and axonal guidance. Each horizontal line indicates the expression pattern of each gene and the vertical columns indicate the uninjured control and time points after SCI. The color chart indicates mean fold change of gene expression in each time points. Red and green colors represent increased and decreased expression respectively.
(JPG)

Figure S8 **Differentially expressed genes involved in different signaling pathways in zebrafish spinal cord after injury.** A–E) Dendrograms representing genes related to BMP/TGFβ signaling, JAK/STAT signaling, Shh signaling, Notch signaling and FGF signaling respectively. Each horizontal line indicates the expression pattern of each gene and the vertical columns indicate the uninjured control and time points after SCI. The color chart indicates mean fold change of gene expression in each time points. Red and green colors represent increased and decreased expression respectively.
(JPG)

Figure S9 Differential expression pattern of genes involved in Ion channel transport, One carbon folate metabolism and N-glycan biosynthesis pathway during regeneration of zebrafish spinal cord.
(JPG)

Figure S10 A) Adult zebrafish showing the dorsal fin level where a wound has been made. B) Inside wound showing uninjured spinal cord, spinal cord after giving control crush injury. C) Skeletal preparation of adult zebrafish stained with Alcian Blue and Alizarine Red, where vertebrae at dorsal fin level are clearly visible. D) Skeletal preparation of adult zebrafish after crush injury in spinal cord at dorsal fin level showing the injured vertebra.
(JPG)

Table S1 **List of genes differentially expressed during zebrafish spinal cord regeneration.**
(XLS)

Table S2 **Table representing number of genes, p-value at different time point related to different enriches functional group in** **figure 2A** **.**
(XLS)

Table S3 **Table represents number of genes, p-value at different time point related to different enriched signaling pathways in** **figure 2B** **.**
(XLS)

Table S4 **List of differentially expressed genes related to inflammation regulation, MHC molecules and complement factors after SCI in zebrafish.**
(XLS)

Table S5 **Table represents quantification of STAT-3 positive cells along with different markers.**
(JPG)

Table S6 **List of differentially expressed genes related to M1 and M2 type macrophages after SCI in zebrafish.**
(XLS)

Table S7 **List of differentially expressed genes related to cell death and anti-apoptosis after SCI in zebrafish.**
(XLS)

Table S8 **List of differentially expressed genes related to cell migration after SCI in zebrafish.**
(XLS)

Table S9 **List of differentially expressed genes related to cellular dedifferentiation process after SCI in zebrafish.**
(XLS)

Table S10 **List of differentially expressed genes related to cell cycle and cell proliferation regulation after SCI in zebrafish.**
(XLS)

Table S11 **List of differentially expressed genes related to neurogenesis and neuronal differentiation after SCI in zebrafish.**
(XLS)

Table S12 **Table represents quantification of SOX2 positive cells along with different markers.**
(JPG)

Table S13 **List of differentially expressed genes related to anterior-posterior and dorso-ventral pattern formation after SCI in zebrafish.**
(XLS)

Table S14 **List of differentially expressed genes related to axonogenesis and axonal guidance after SCI in zebrafish.**
(XLS)

Table S15 **List of differentially expressed genes related to different signaling pathways after SCI in zebrafish.**
(XLS)

Table S16 **List of differentially expressed genes commonly expressed in fin, retina, heart and spinal cord regeneration in zebrafish.**
(XLS)

Table S17 **List of differentially expressed genes related to N-glycan biosynthesis, one carbon folate metabolism and ion channel transport after SCI in zebrafish.**
(XLS)

Table S18 **List of differentially expressed unannotated genes after SCI in zebrafish.**
(XLS)

Table S19 **List of primers, Tm, primer annealing time and product length of annotated genes used for qRT-PCR.**
(JPG)

Table S20 **List of primers, Tm, primer annealing time and product length of unannotated genes used for qRT-PCR.**
(JPG)
