# Supplementary figures and images for: Genome Wide Expression Profiling during Spinal Cord Regeneration Identifies Comprehensive Cellular Responses in Zebrafish
Source: PLoS One. 2014 Jan 20;9(1):e84212. doi: 10.1371/journal.pone.0084212 (PMC3896338; doi:10.1371/journal.pone.0084212)

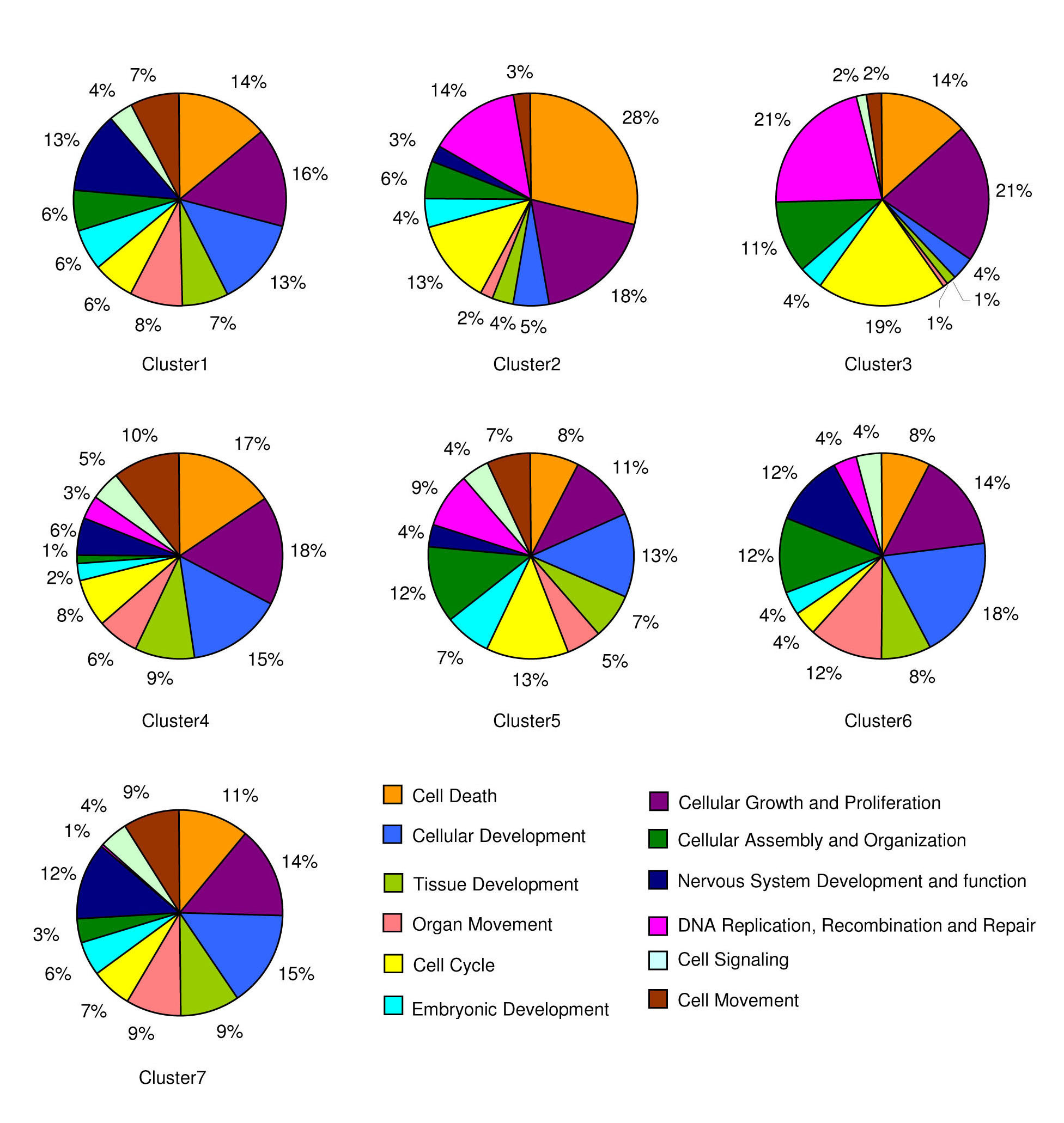

Supplement: Figure S1 — Pie charts showing the percentage of functionally enriched genes that are differentially expressed in the seven clusters. (JPG) [file pone.0084212.s001.jpg]

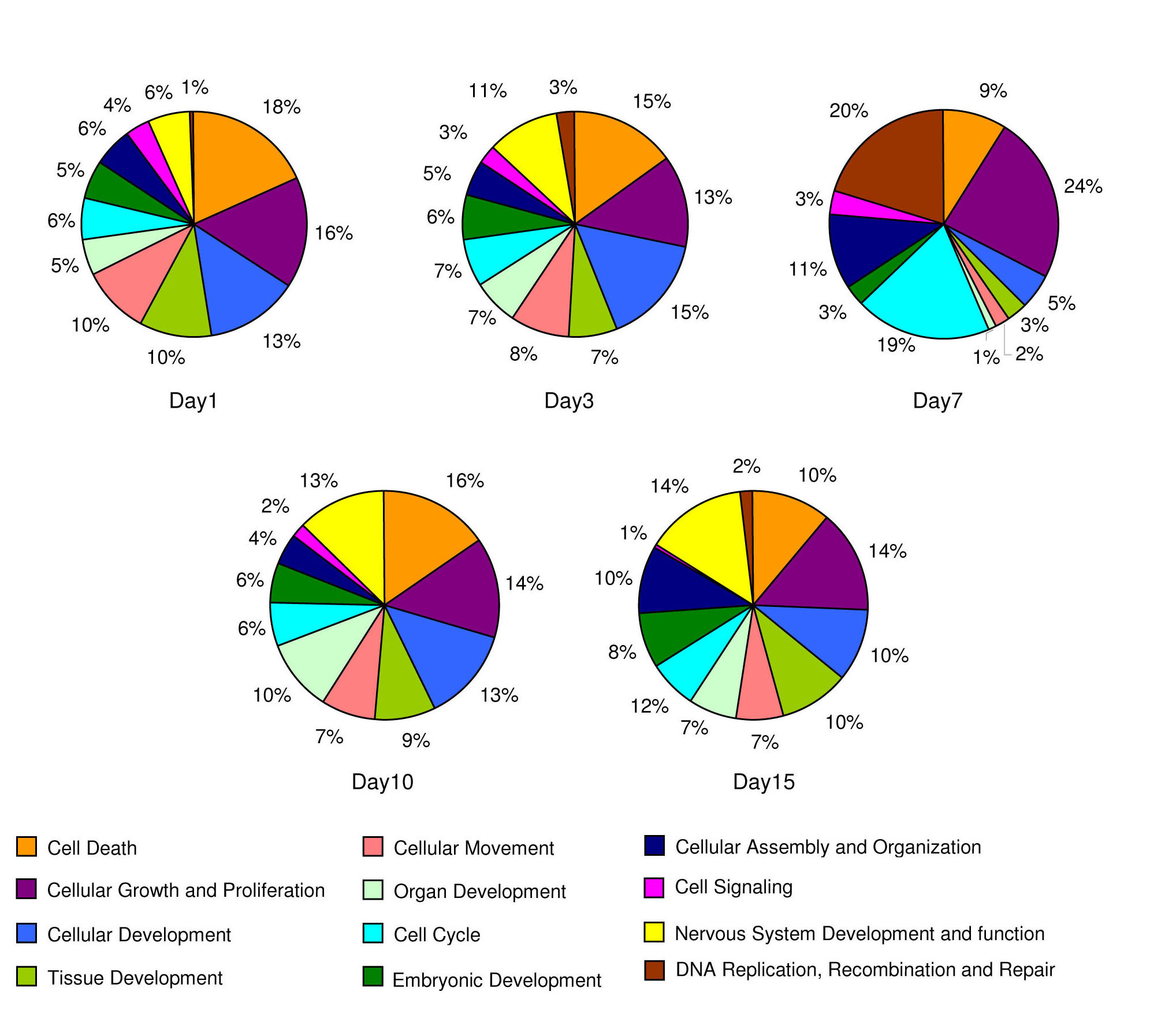

Supplement: Figure S2 — Pie charts show the percentage of functionally enriched genes that are differentially expressed at different time points after SCI in zebrafish. (JPG) [file pone.0084212.s002.jpg]

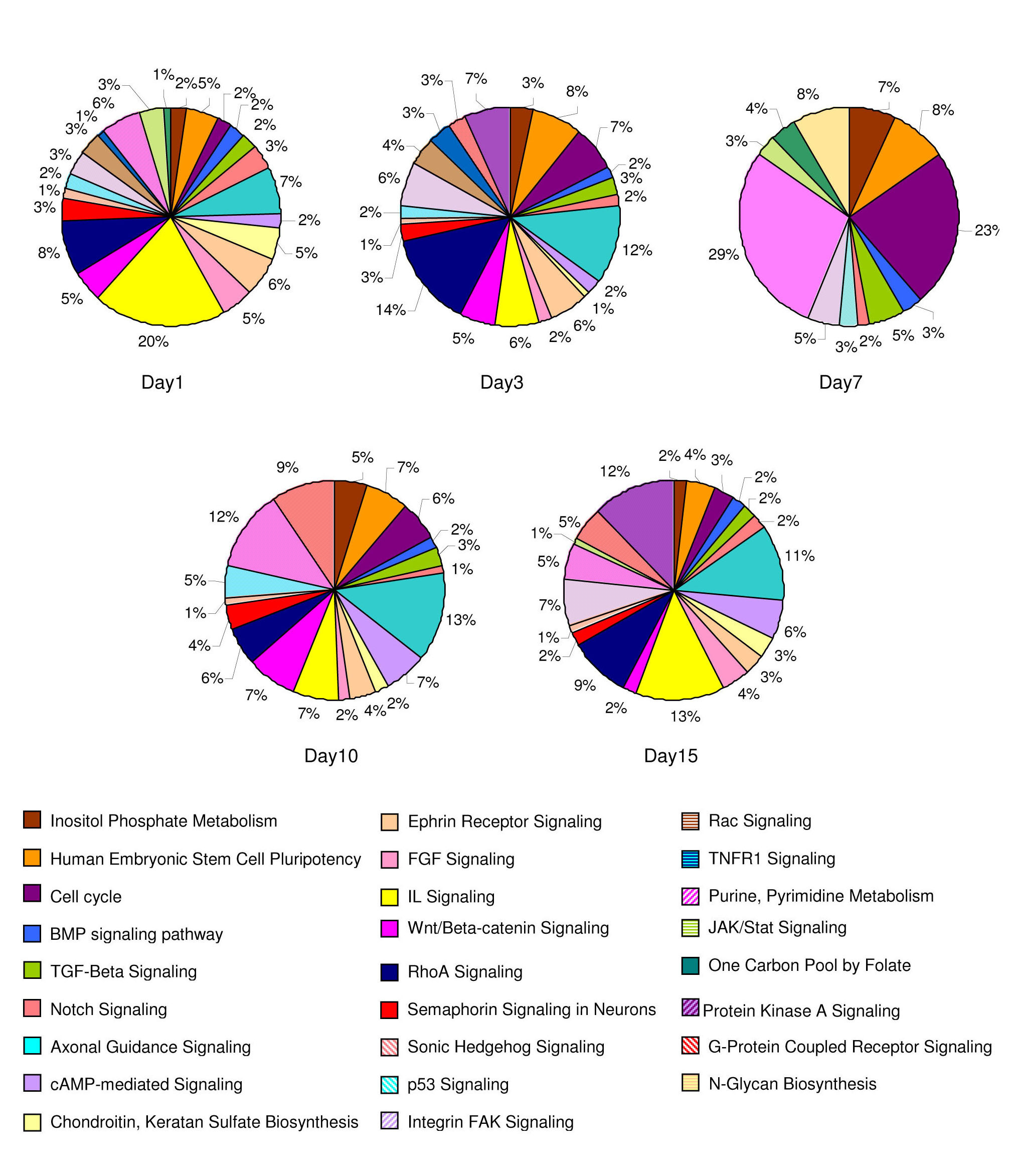

Supplement: Figure S3 — Pie charts showing the percentage of canonical pathway enriched genes that are differentially expressed at different time points after SCI in zebrafish. (JPG) [file pone.0084212.s003.jpg]

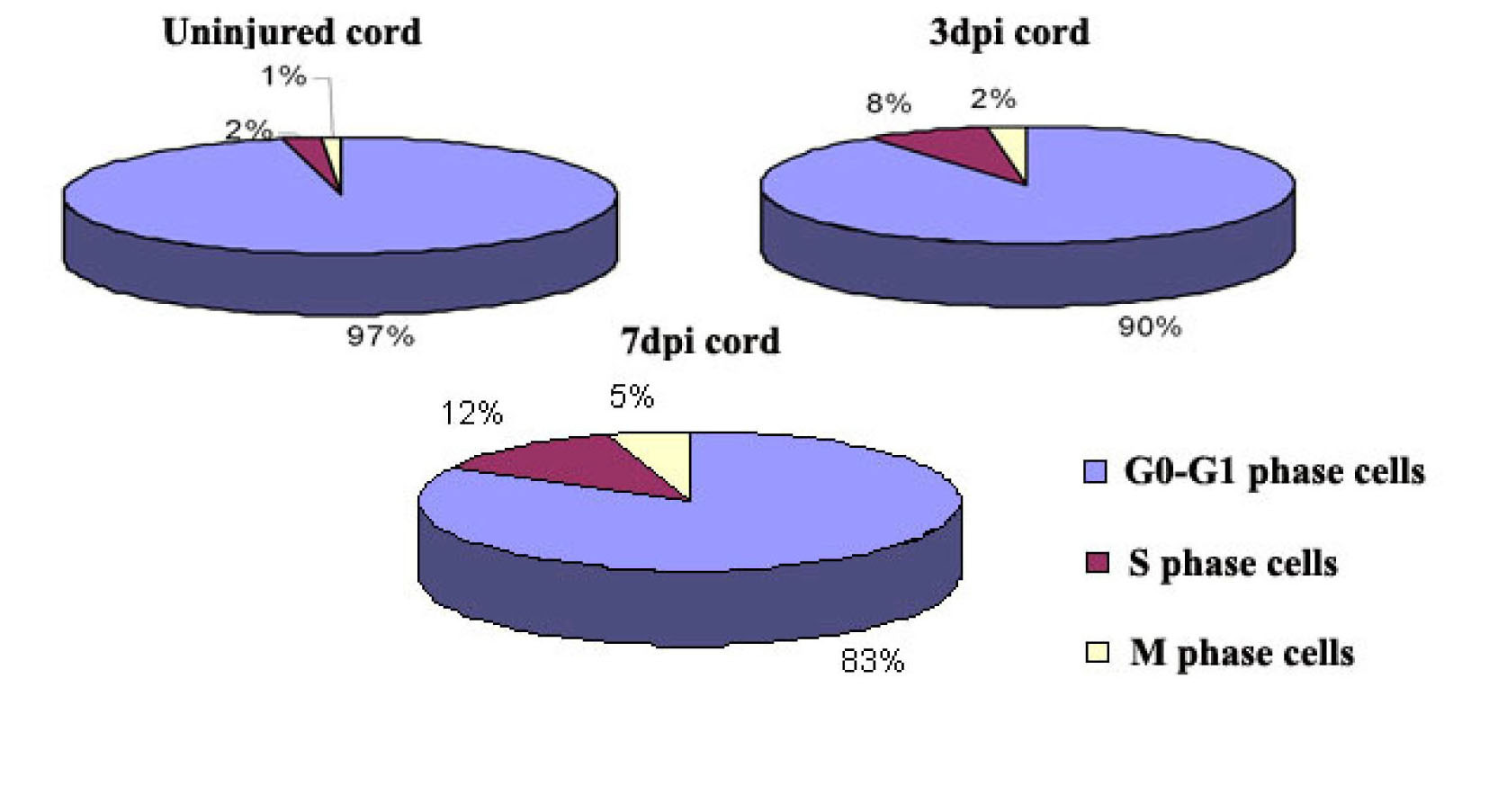

Supplement: Figure S4 — Quantification of cells present in different cell cycle phases in uninjured and injured cord based on BrdU, H3P and DAPI colocalization study. A) In uninjured cord only 2% and 1% of cells are in S-phase and M-phase respectively. B) In 3 dpi cord the percentage of S-phase cells have been increased significantly to 8% than uninjured cord and only 2% cells are in M-phase. C) In 7 dpi cord 12% of total populations are in S-phase and 5% of total populations are in M-phase. (JPG) [file pone.0084212.s004.jpg]

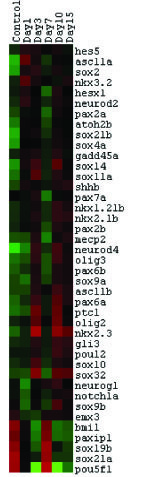

Supplement: Figure S5 — Dendrogram represents differential expression pattern of transcription factors involved in neurogenesis and neuronal specification during regeneration of zebrafish spinal cord. Each horizontal line indicates the expression pattern of each gene and the vertical columns indicate the uninjured control and time points after SCI. The color chart indicates mean fold change of gene expression in each time points. Red and green color represents increased and decreased expression respectively. (JPG) [file pone.0084212.s005.jpg]

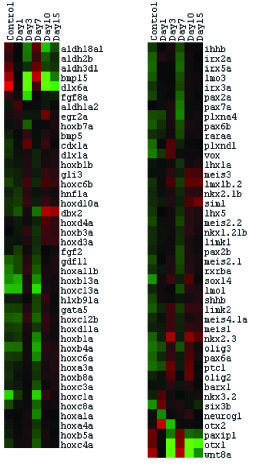

Supplement: Figure S6 — Differentially expressed genes related to pattern formation are represented in two dendograms in regenerating zebrafish spinal cord. Each horizontal line indicates the expression pattern of each gene and the vertical columns indicate the uninjured control and time points after SCI. The color chart indicates mean fold change of gene expression in each time points. Red and green colors represent increased and decreased expression respectively. (JPG) [file pone.0084212.s006.jpg]

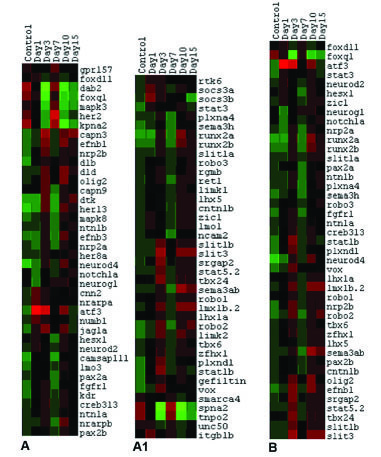

Supplement: Figure S7 — Differentially expressed genes related to axonogenesis and axonal guidance is represented in two different dendrograms (A and A1) in regenerating zebrafish spinal cord. Dendrogram (B) represents differential expression pattern of transcription factors involved in axonogenesis and axonal guidance. Each horizontal line indicates the expression pattern of each gene and the vertical columns indicate the uninjured control and time points after SCI. The color chart indicates mean fold change of gene expression in each time points. Red and green colors represent increased and decreased expression respectively. (JPG) [file pone.0084212.s007.jpg]

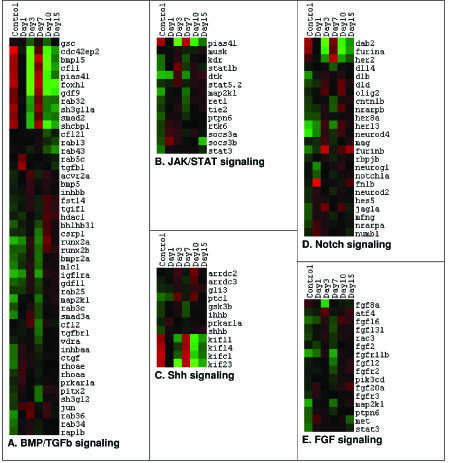

Supplement: Figure S8 — Differentially expressed genes involved in different signaling pathways in zebrafish spinal cord after injury. A–E) Dendrograms representing genes related to BMP/TGFβ signaling, JAK/STAT signaling, Shh signaling, Notch signaling and FGF signaling respectively. Each horizontal line indicates the expression pattern of each gene and the vertical columns indicate the uninjured control and time points after SCI. The color chart indicates mean fold change of gene expression in each time points. Red and green colors represent increased and decreased expression respectively. (JPG) [file pone.0084212.s008.jpg]

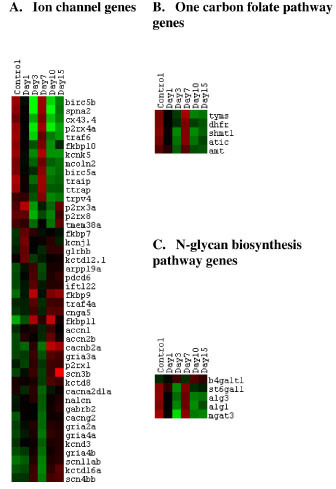

Supplement: Figure S9 — Differential expression pattern of genes involved in Ion channel transport, One carbon folate metabolism and N-glycan biosynthesis pathway during regeneration of zebrafish spinal cord. (JPG) [file pone.0084212.s009.jpg]

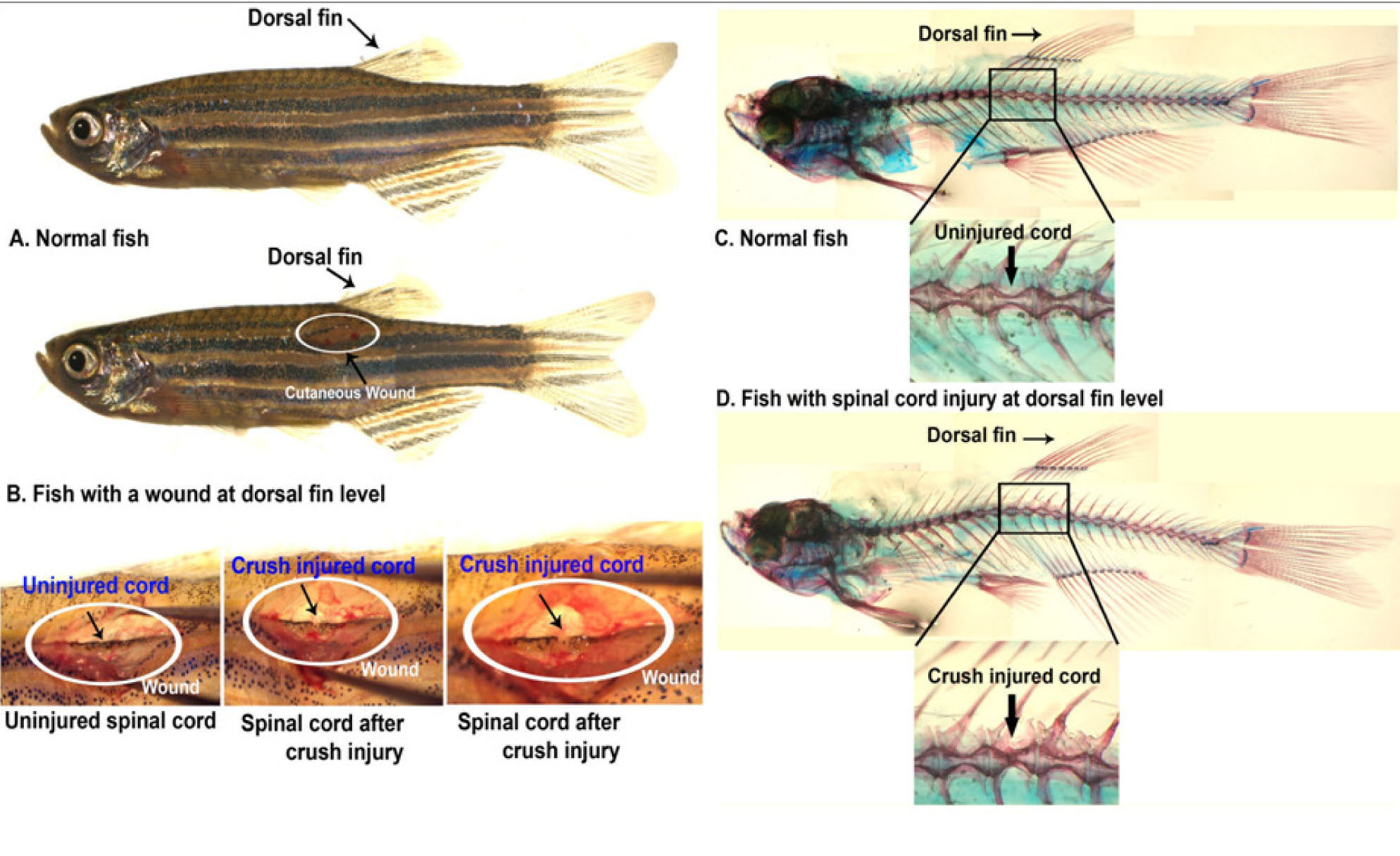

Supplement: Figure S10 — A) Adult zebrafish showing the dorsal fin level where a wound has been made. B) Inside wound showing uninjured spinal cord, spinal cord after giving control crush injury. C) Skeletal preparation of adult zebrafish stained with Alcian Blue and Alizarine Red, where vertebrae at dorsal fin level are clearly visible. D) Skeletal preparation of adult zebrafish after crush injury in spinal cord at dorsal fin level showing the injured vertebra. (JPG) [file pone.0084212.s010.jpg]

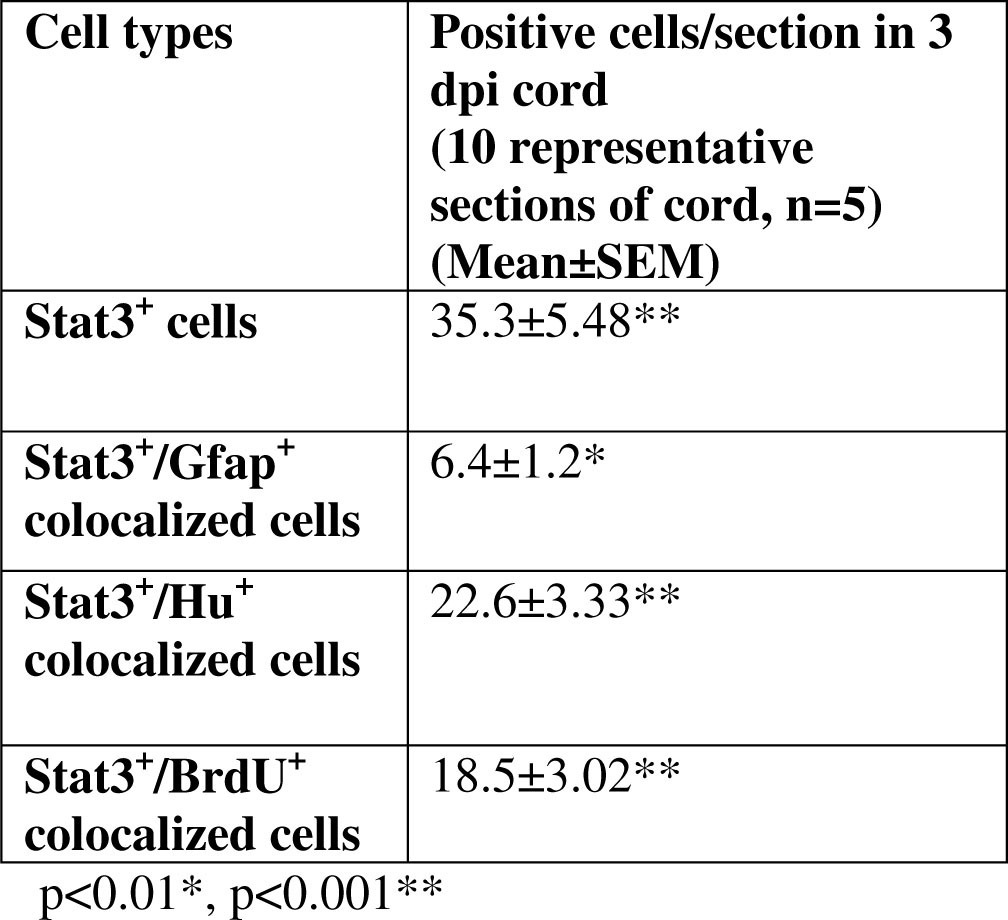

Supplement: Table S5 — Table represents quantification of STAT-3 positive cells along with different markers. (JPG) [file pone.0084212.s015.jpg]

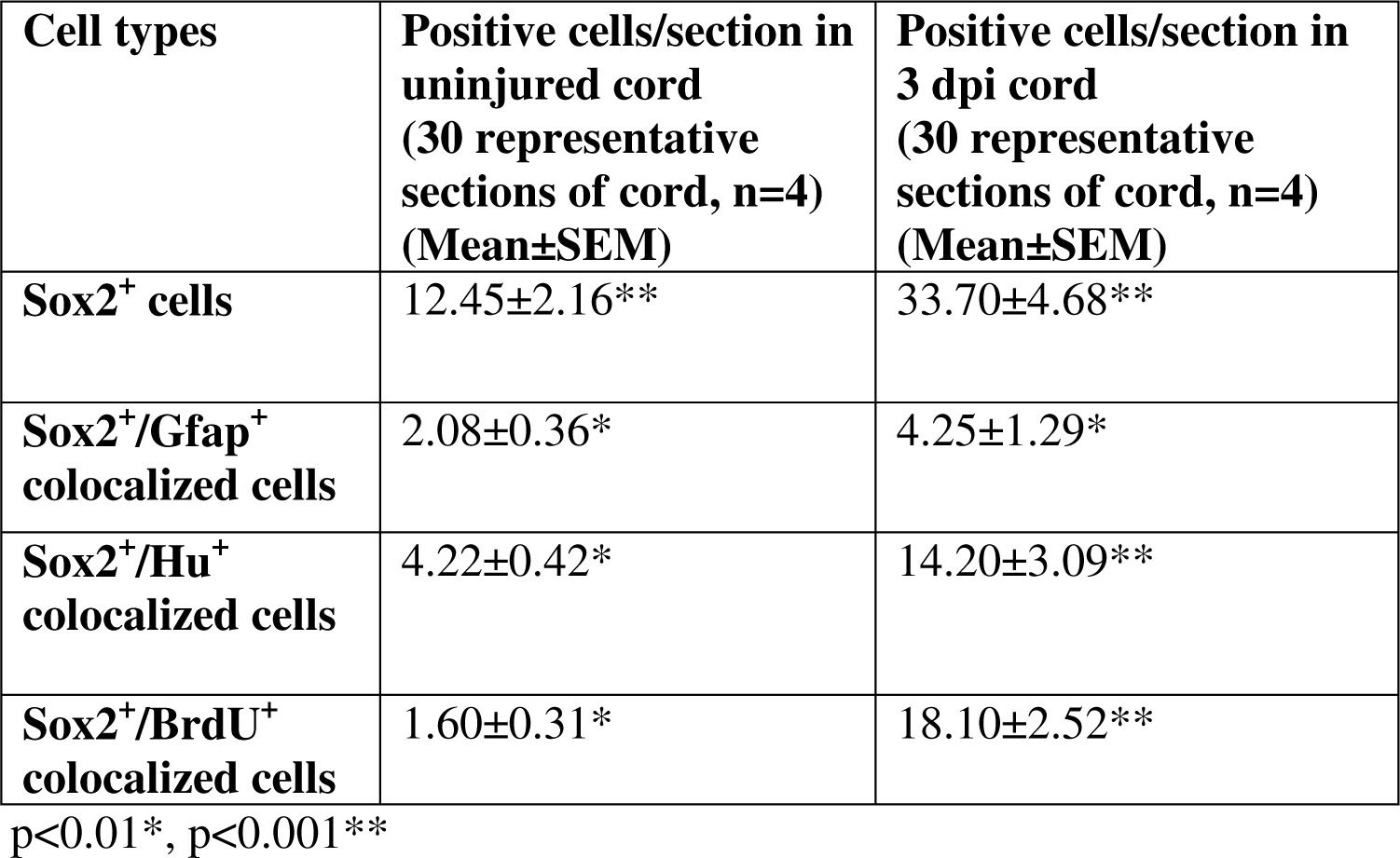

Supplement: Table S12 — Table represents quantification of SOX2 positive cells along with different markers. (JPG) [file pone.0084212.s022.jpg]

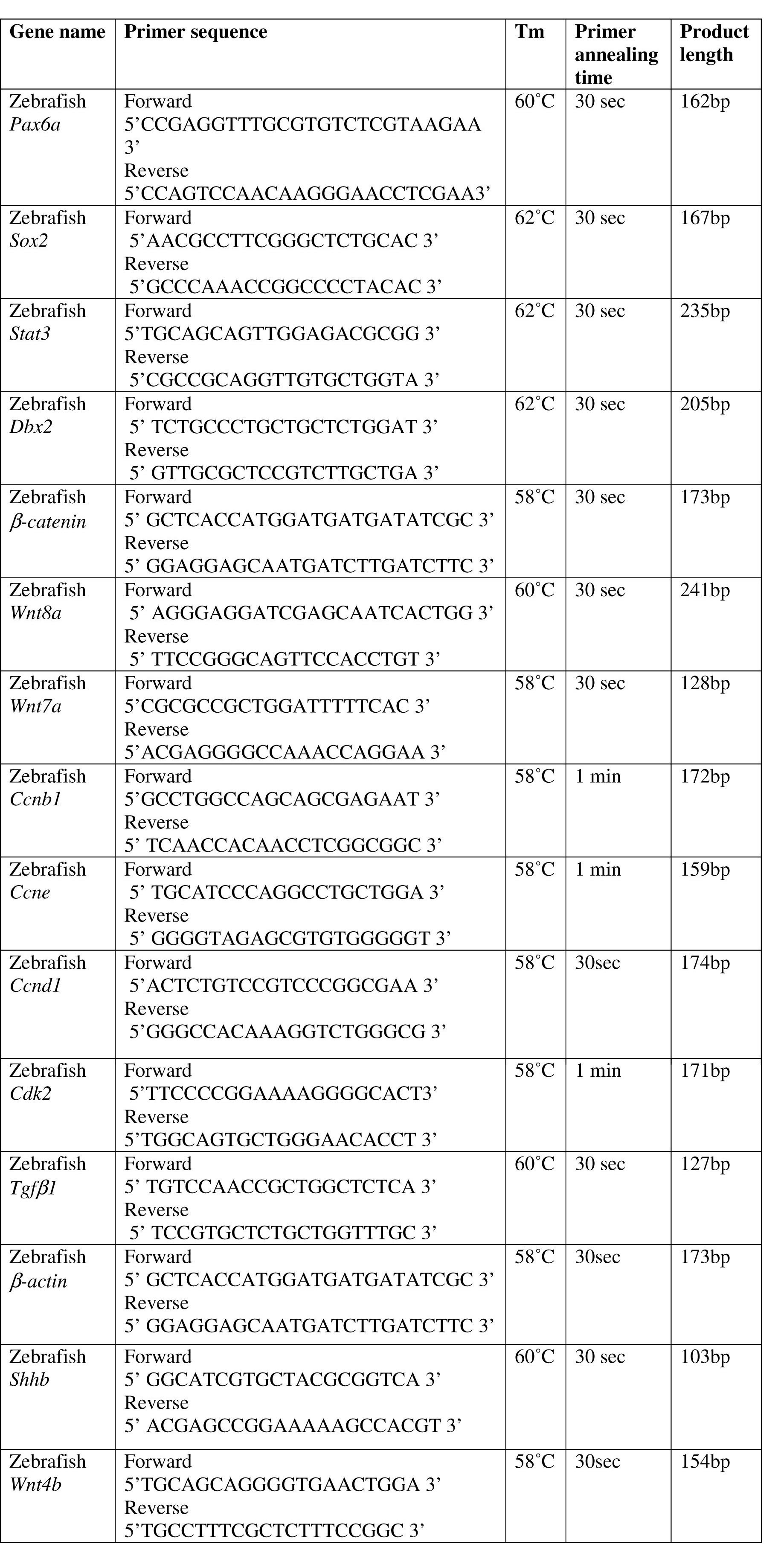

Supplement: Table S19 — List of primers, Tm, primer annealing time and product length of annotated genes used for qRT-PCR. (JPG) [file pone.0084212.s029.jpg]

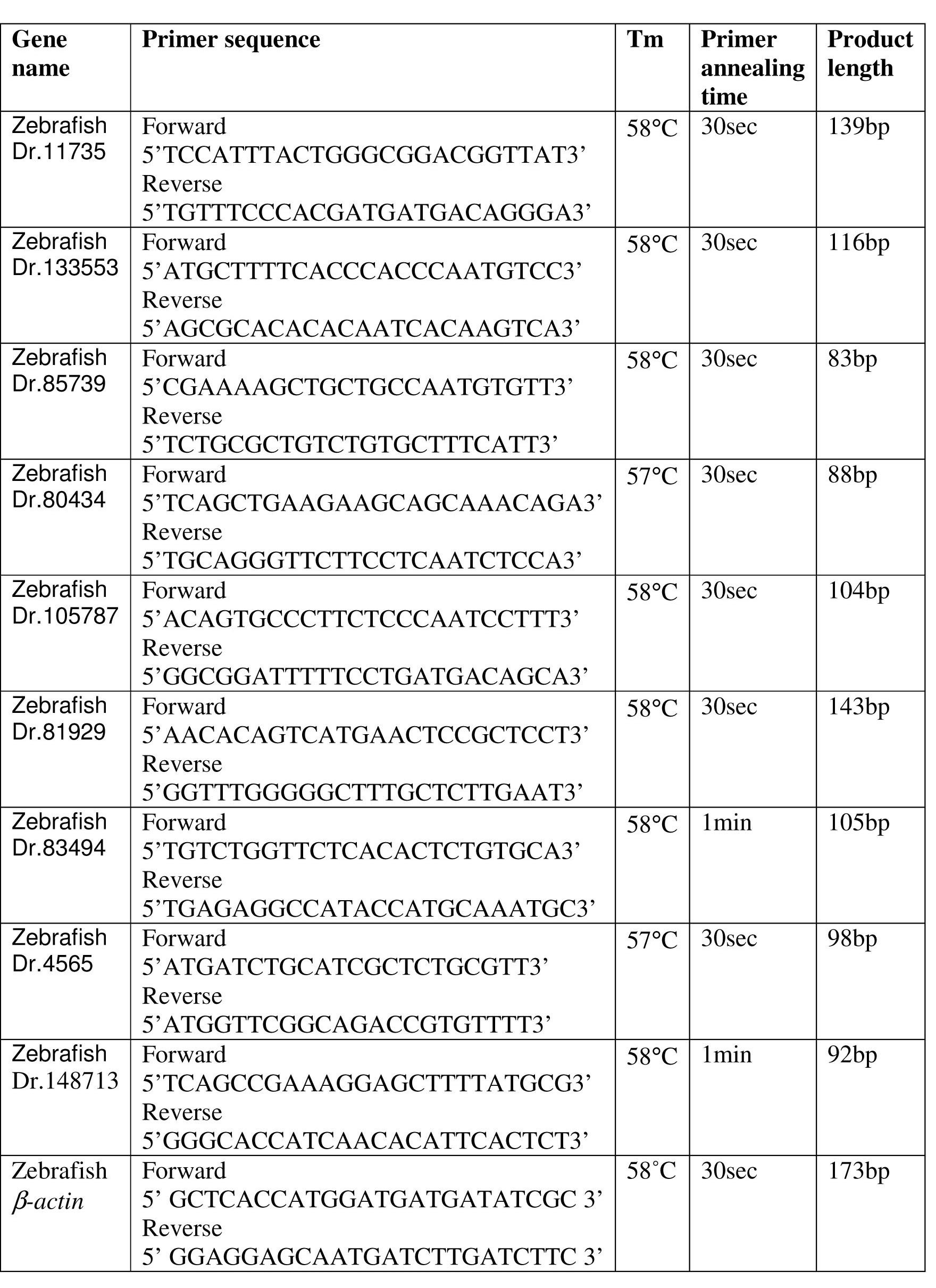

Supplement: Table S20 — List of primers, Tm, primer annealing time and product length of unannotated genes used for qRT-PCR. (JPG) [file pone.0084212.s030.jpg]
